# Dietary Restriction during Development Enlarges Intestinal and Hypodermal Lipid Droplets in *Caenorhabditis elegans*

**DOI:** 10.1371/journal.pone.0046198

**Published:** 2012-11-20

**Authors:** Daniela Palgunow, Maja Klapper, Frank Döring

**Affiliations:** Department of Molecular Prevention, Institute of Human Nutrition and Food Science, Christian-Albrechts-University of Kiel, Kiel, Germany; Lancaster University, United Kingdom

## Abstract

Dietary restriction (DR) extends lifespan in man species and modulates evolutionary conserved signalling and metabolic pathways. Most of these studies were done in adult animals. Here we investigated fat phenotypes of *C. elegans* larvae and adults which were exposed to DR during development. This approach was named “developmental-DR” (dDR). Moderate as well as stringent dDR increased the triglyceride to protein ratio in L4 larvae and adult worms. This alteration was accompanied by a marked expansion of intestinal and hypodermal lipid droplets. In comparison to *ad libitum* condition, the relative proportion of fat stored in large lipid droplets (>50 µm^3^) was increased by a factor of about 5 to 6 in larvae exposed to dDR. Microarray-based expression profiling identified several dDR-regulated genes of lipolysis and lipogenesis which may contribute to the observed fat phenotypes. In conclusion, dDR increases the triglyceride to protein ratio, enlarges lipid droplets and alters the expression of genes functioning in lipid metabolism in *C. elegans*. These changes might be an effective adaptation to conserve fat stores in animals subjected to limiting food supply during development.

## Introduction

Dietary restriction (DR) also known as caloric restriction (CR) is defined as a significant reduction of energy and macronutrient intake in the absence of malnutrition [1]. In adult animals, DR is a fundamental nutritional intervention to reduce body weight and to decrease the risk of common diseases such diabetes type 2 or atherosclerosis [2], [3]. It extends lifespan and health-span in many species, ranging from invertebrates to mammals [3]. In recent years, the short-lived model organisms *Saccharomyces cerevisiae*, *Drosophila melanogaster* and *Caenorhabditis elegans* were used to uncover key regulatory factors mediating DR induced longevity. These include the target of rapamycin TOR [4]–[6], the AMP-activated protein kinase AMPK [7] and the Insulin/IGF-1 signaling cascade [8]. In *Drosophila*, insulin-like signalling converges with DR and seems to be a downstream target of DR [9]. In *C. elegans*, insulin/IGF-1 signaling and DR seem to be independent pathways in regulating lifespan [7], [10]–[12]. In addition, several transcription factors including the Forkhead transcription factor *pha-4* [13], the Nrf2 transcription factor *skn-1* [14] and the heat-shock transcription factor *hsf-1* [15] were recognized as important regulators mediating longevity by DR in *C. elegans*. Of note, most of these pathways were identified in adult animals.

During the last years, *C. elegans* has been emerged as an important model to study the regulation of energy metabolism and lipid storage. As a great advantage, it enables the examination of the relationship of lipid metabolism, growth, reproduction and lifespan. Many of mammalian metabolic pathways, such as fatty acid (FA) synthesis, elongation and desaturation, mitochondrial and peroxisomal ß-oxidation of fatty acids are conserved in the nematode. A number of genes involved in pathways that regulate lipid homeostasis in mammals are assumed to control lipid storage as well in *C. elegans*. These include serotonin, insulin, transforming growth factor-ß (TGF-ß) and TOR signalling pathways [16]–[20]. In addition, many mammalian transcription factors involved in fat accumulation are present in *C. elegans*. For example, the *C. elegans* transcription factors SBP-1 and NHR-49 are homologues to the human sterol-regulatory-element-binding protein (SREBP) and peroxisome proliferator-activated receptor-α (PPARα), respectively [18], [21]–[23]. Thus, the worm seems to be an appropriate model to study lipid metabolism. However, only few studies have investigated the influence of DR on *C. elegans* fat phenotypes. For example, DR in liquid medium or dietary deprivation results in a pale appearance of adult worms suggesting a mobilization of intestinal fat stores [14], [21].

A variety of protocols exist to subject *C. elegans* to DR. In the laboratory, *C. elegans* is usually fed on *Escherichia coli* (OP50) lawns cultivated on Nematode Growth Medium (NGM) agar plates. One common strategy to generate DR is the limitation of *E. coli* growth on solid medium by UV, heat or antibiotic treatment. The reduction of bactopeptone in the NGM agar plates is another strategy to control growth of *E. coli* [24]–[26]. Cultivation of *C. elegans* on plates in the absence of bacteria (dietary deprivation) is usually performed during adulthood [15], [27], [28]. In liquid media, DR is induced by dilution of *E. coli* in S-Basal medium [11], [13], [14], [29], undefined axenic medium [30] and chemically defined liquid medium [31]. The use of feeding defective mutants such as *eat-2* is a further approach to study DR in *C. elegans* [10]. However, all of these methods were primary established to examine effects of DR during adulthood.

Most studies performing DR in *C. elegans* have examined the effects and molecular mechanisms of DR on lifespan extension in adult worms. So far, effects of DR applied during development have not been extensively analyzed in *C. elegans*. Here, we investigated in *C. elegans* the impact of DR during development. For this purpose, we have optimized a solid medium based DR protocol [24], [32], [33], in which bactopeptone is excluded from the agar plates and the addition of antibiotics is not required. This DR regime was named “developmental-DR” (dDR). It increased the triglyceride to protein ratio and lipid droplet (LD) size in larvae and adult worms exposed to DR during development. To gain an insight into underlying mechanisms of dDR induced phenotypes, whole genome gene expression analysis was performed. Among the dDR responsive genes we found an enrichment of genes being involved in lipid metabolism. These genes might contribute to dDR induced fat phenotypes.

## Results

### Establishment of a dietary restriction (DR) protocol (developmental-DR, dDR) which allows the application of DR during development from hatching to adulthood

**Figure 1:**
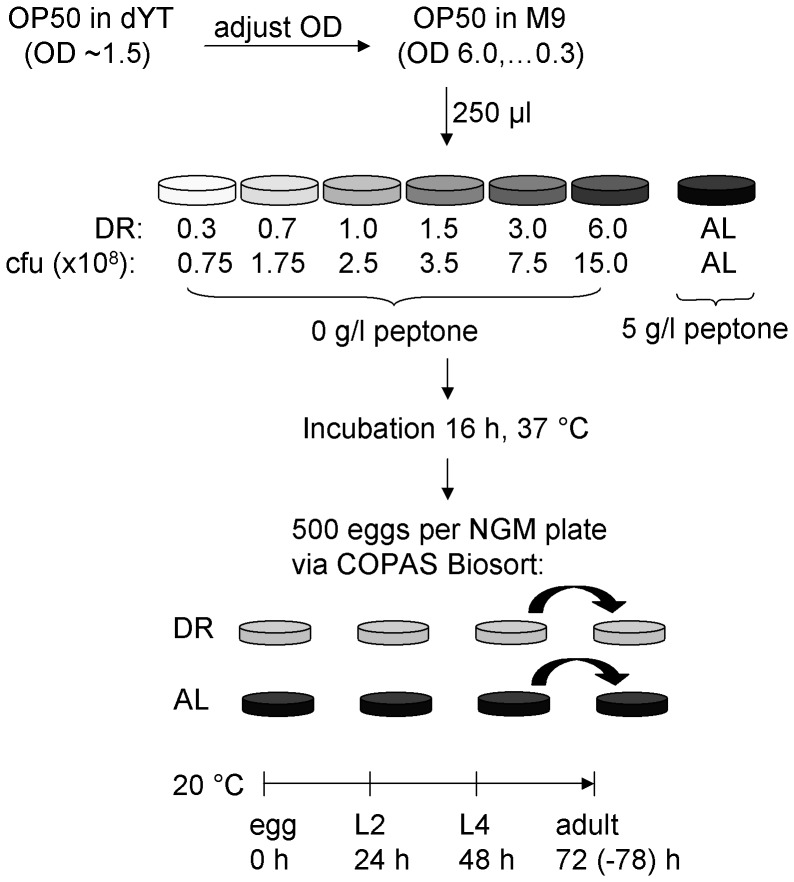
Scheme of the established solid medium based dietary restriction method to apply dietary restriction during development (dDR) in *C. elegans*. *Escherichia coli* (*E.* coli) OP50, serving as food source for *C. elegans*, was cultivated at 37°C until an optical density (OD_600_) = 1.5 was reached (OD_600_ = 1.0≈1.0×10^9^ cfu/ml). Different ODs (6.0, 3.0, 1.5, 1.0, 0.7, 0.3) were adjusted in M9 buffer. Standard Nematode Growth Medium (NGM) was used for *ad libitum* (AL) condition. For dietary restriction (dDR), bactopeptone was omitted from NGM agar plates to prevent bacterial growth. A defined amount (250 µl) of different OP50 suspensions was seeded on dDR plates, resulting in a gradient of colony forming units (cfu) per plate. For AL plates, an OP50 suspension of OD1.5 was used. Nematodes were synchronized by hypochlorite treatment, and 500 eggs per agar plate were sorted via cytometry-based COPAS Biosort system. Animals were cultivated at 20°C and harvested at second larval stage (L2, 24 h after hatching), fourth larval stage (L4, 48 h) or after reaching adulthood (adult, 72–78 h). At L4 stage, nematodes were transferred once to fresh agar plates.

Based on reported DR methods [24], [32], here we established a modified solid medium based DR protocol in order to study the effect of DR on *C. elegans* larvae and adults exposed to DR during development (**Figure 1**). This method was named “developmental dietary restriction” (dDR). It allows a standardized variation of the extent of dDR without starvation, dauer formation or arrest of the animals during development. In brief, adjusted optical densities (250 µl, OD_600_ 0.3 to 6.0, represents DR 0.3 to 6.0) of the *E. coli* strain OP50 were spread onto antibiotic-free agar plates and incubated for 16 h at 37°C. For dDR conditions, bactopeptone as sole carbon source for bacterial growth was omitted from the Nematode Growth Medium (NGM). This leads to different amounts of bacteria per agar plate depending on the OD of seeded OP50 (e. g. dDR 1.5: 0.56±0.05 OD/plate; dDR 0.7: 0.32±0.03 OD/plate). For *ad libitum* (AL) condition, standard (NGM) was used resulting in a thick bacteria lawn (25.85 (mean)±1.19 (SD) OD/plate). To standardize food availability per worm, 500 synchronized embryos were sorted onto AL and dDR agar plates by flow cytometry and were cultivated at 20°C until reaching the L2, L4 or adult stage. To exclude starvation, L4 larvae and adult worms were transferred daily to fresh agar plates.

### dDR reduces body size without substantial changes of developmental time, locomotion and feeding rate

**Figure 2:**
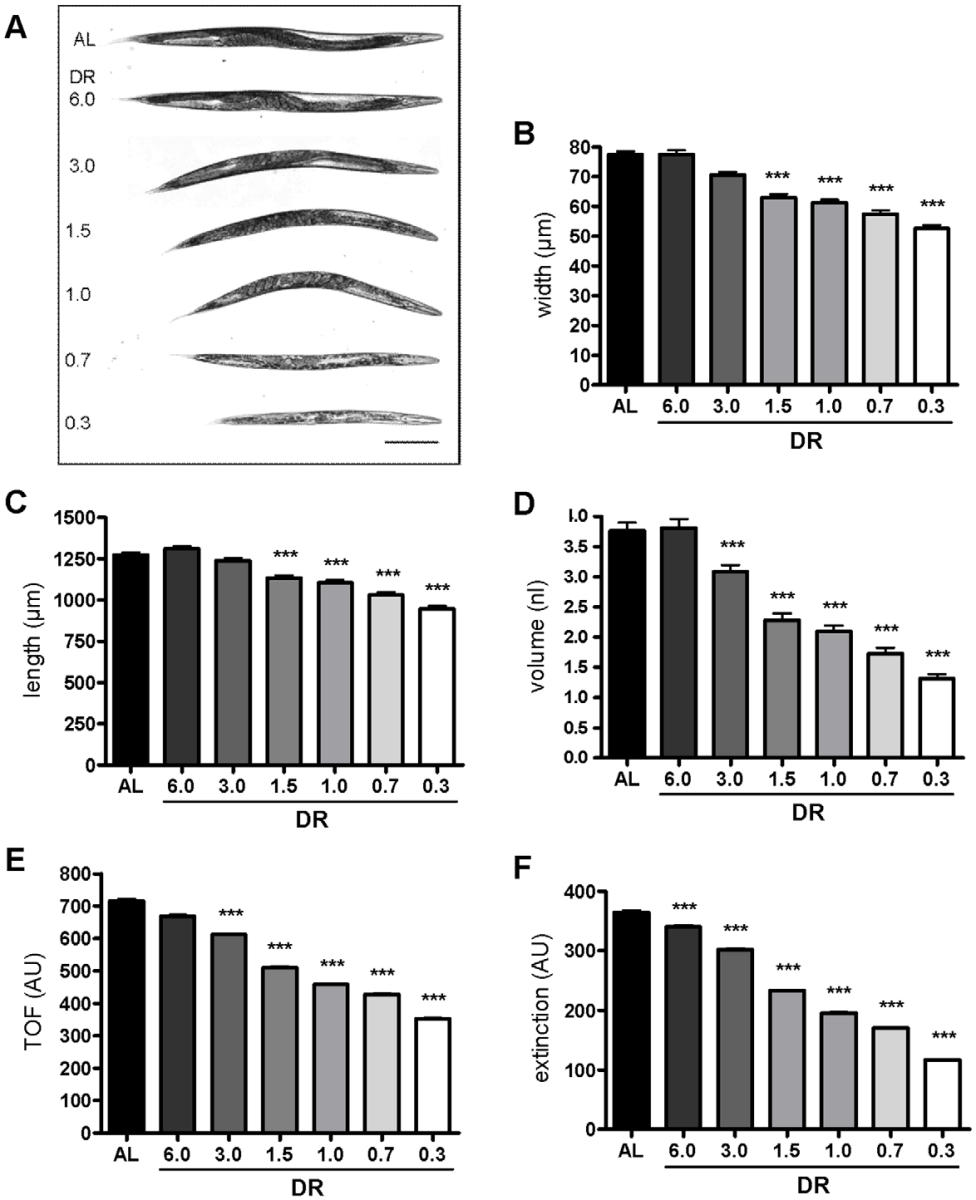
Influence of dDR on body proportion. Wild-type nematodes were cultivated under AL or specified dDR condition and analyzed at first day of adulthood. (A) Representative bright-field microscopy photographs of nematodes (n>50 per condition) in dependence on OP50 density. The anterior part is on the right. Magnification 50×; scale bar, 200 µm. (B–D). Data for body width (µm), length (µm) and volume (nl) of single worms (n>50 per condition) derived from bright-field microscopy images. The body volume was calculated using a worm adapted cylinder volume formula which includes area and perimeter of single animals. Comparative calculations between AL and dDR treated animals are provided in **Table S1**. (E, F) Time of flight (TOF, AU) and extinction (AU) values (n>500 per condition) were collected by COPAS Biosort system. Bars represent a mean ± SEM from two to three independent experiments (***p<0.001). AU = arbitrary unit.

To evaluate our dDR protocol, body proportions of adult worms exposed to DR during development were determined based on bright-field microscopy images (**Figure 2A–D** **, Table S1**). As expected, we obtained an inverse relationship between the extent of dDR (0.3 to 6.0) and reduction of body width (**Figure 2B**), length (**Figure 2C**) and volume (**Figure 2D**) of adult worms. This demonstrates dose dependency of our dDR regimes. Interestingly, the body width decreased to a greater extent than the length (**Table S1**). At the most stringent dDR condition (dDR0.3), width declined to approximately 68%, whereas length was reduced to about 74% when compared with AL fed worms. The body volume of dDR0.3 fed worms (1.3 nl) decreased by a factor of 3 (34.8±2.1% of AL). Flow cytometry based TOF (time of flight) and extinction (ext) values, which serve as proxies for length and volume of the worms [34], confirmed the microscopic body proportion measurements in a large number of worms (**Figure 2E/F**). Because dDR0.7 and dDR1.5 did not substantial influence the time point of the first egg lay (AL: ∼72 h; dDR1.5: ∼74 h; dDR1.5: ∼76 h), locomotion (**Figure S1A–C**) and feeding rate (**Figure S2D**), these conditions were used for further experiments.

### dDR increases the triglyceride to protein ratio in larvae and adult worms

**Figure 3:**
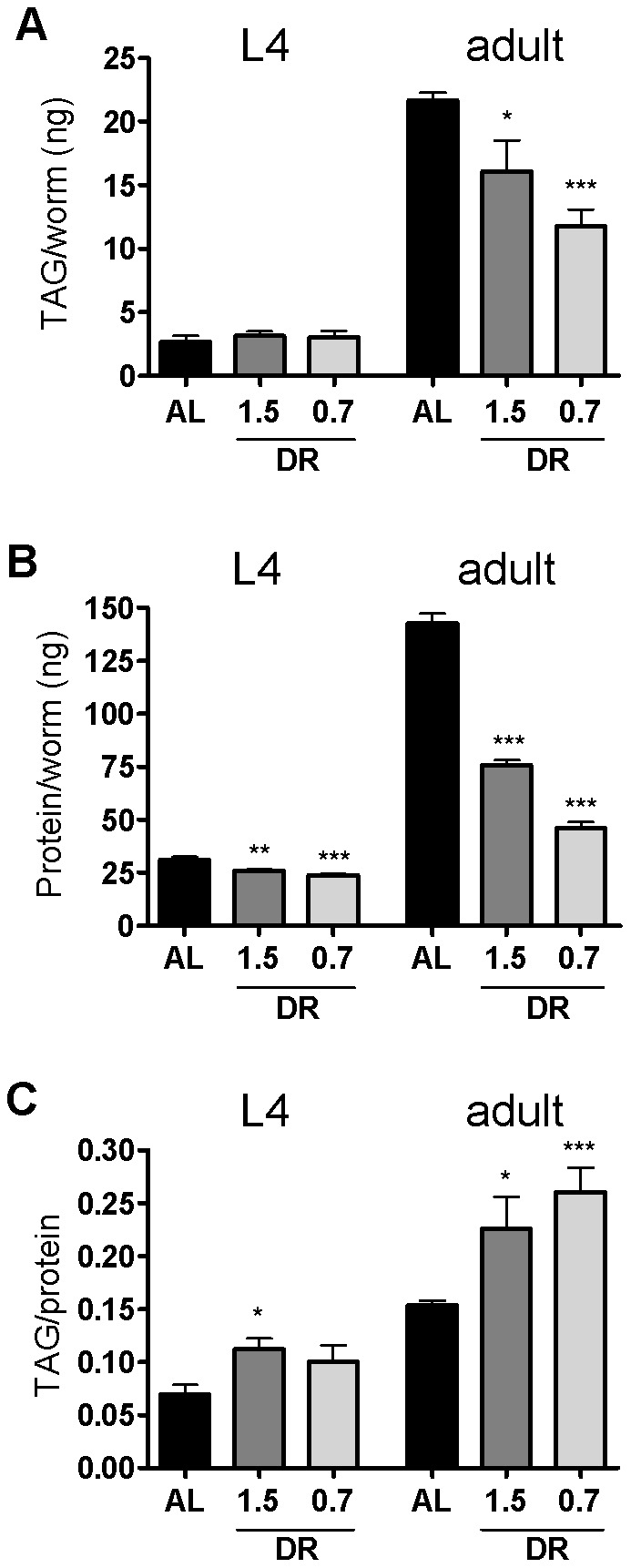
Influence of dDR on triglyceride (TAG) and protein content. (A) TAG content per worm (ng), (B) protein content per worm (ng/w) and (C) TAG/protein ratio of AL and dDR (dDR1.5, dDR0.7) fed wild-type worms were compared. Animals were grown until L4 larval stage or first day of adulthood. Results are represented as mean ± SEM of three independent experiments (*p<0.05, **p<0.005, ***p<0.001).

To get insight into the effect of dDR on body composition triacylglyceride (TAG) and protein levels were determined using an enzymatic assay in L4 and adult worms exposed to DR during development. In L4 larvae and in adulthood, the TAG content per worm was increased, whereas protein content per worm was reduced under moderate and stringent dDR conditions (**Figure 3A/B**). As a consequence, the resulting TAG to protein ratio was increased in L4 larvae and adult worms when compared to control animals (**Figure 3C**). In agreement, thin layer chromatography revealed a higher TAG to phospholipid ratio in adulthood subjected to dDR (**Figure S2**). Thus, biochemical measurements revealed that dDR induced a remarkable shift to a higher TAG to protein ratio in L4 larvae and adult worms.

### dDR increases lipid droplet size in larvae and adult worms

**Figure 4:**
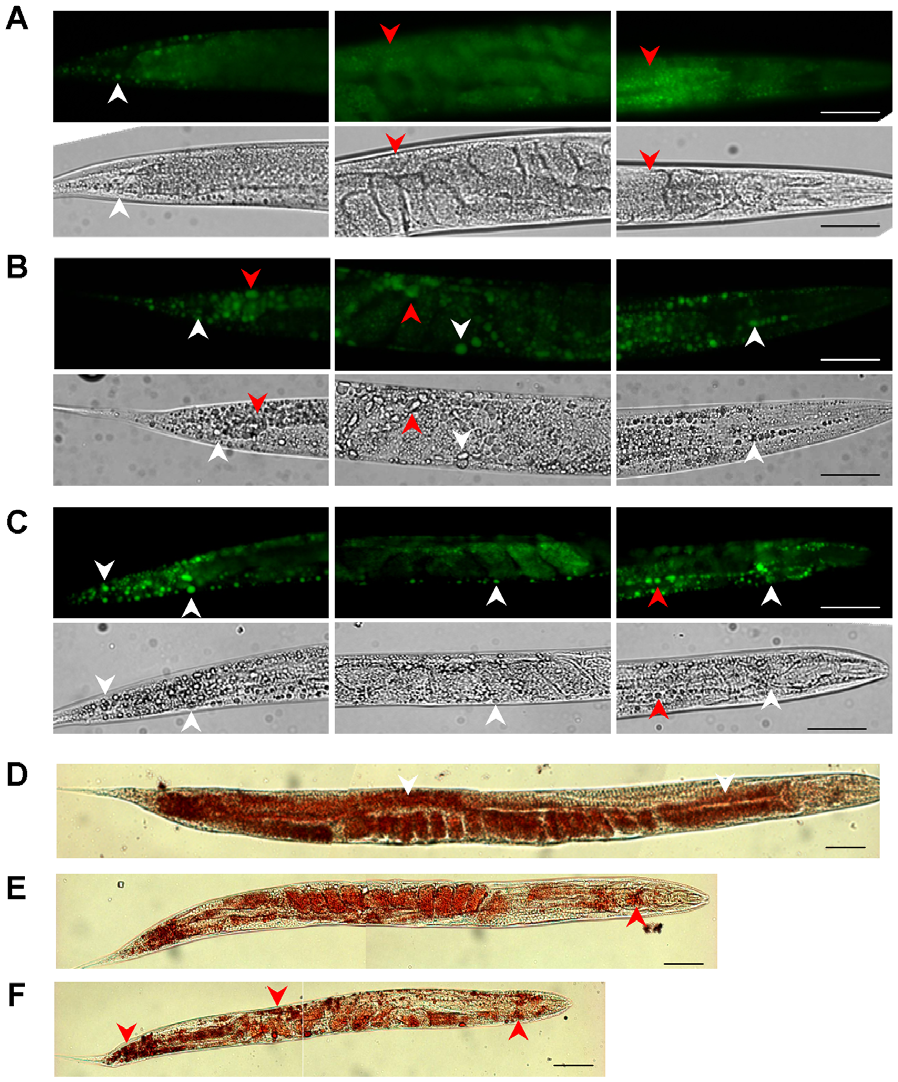
Fat staining of dDR adult *C. elegans*. (A–C) BODIPY 493/503 (fixative staining) fluorescence microscopy and corresponding bright-field microscopy images of AL (A), dDR1.5 (B) and dDR0.7 (C) fed wild-type worms at first day of adulthood. (D–F) Oil red O staining of AL (D), dDR1.5 (E) and dDR0.7 (F) treated adult wild-type worms. Magnification of all photographs 200×; scale bar, 50 µm. The anterior part is on the right. Arrow heads indicate lipid droplets (LD) in the intestine (red) or in the hypodermis (white).

**Figure 5:**
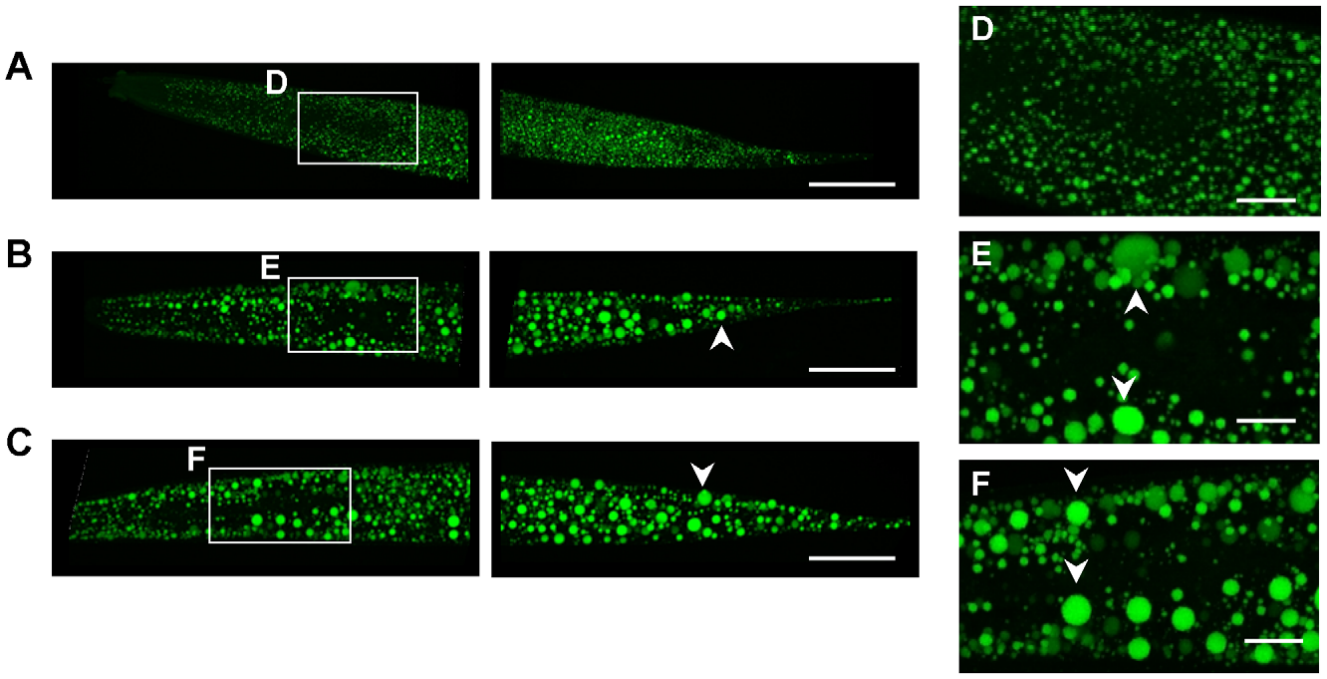
Imaging of lipid droplets in dDR L2 larvae by scanning-laser confocal microscopy. Wild-type animals were cultivated on AL (A), dDR 1.5 (B) and dDR 0.7 (C) agar plates until reaching the L2 stage and harvested for fixative BODIPY 493/503 staining. Images derived from scanning-laser confocal (SLC) microscopy are shown as maximum projection of 20–30 images from a z-stack at 0.5 µm interval. The anterior part is on the left. (D–F) Magnification of LDs in pharynx region. Arrow heads indicate enlarged hypodermal LDs. Magnification 630×.

**Figure 6:**
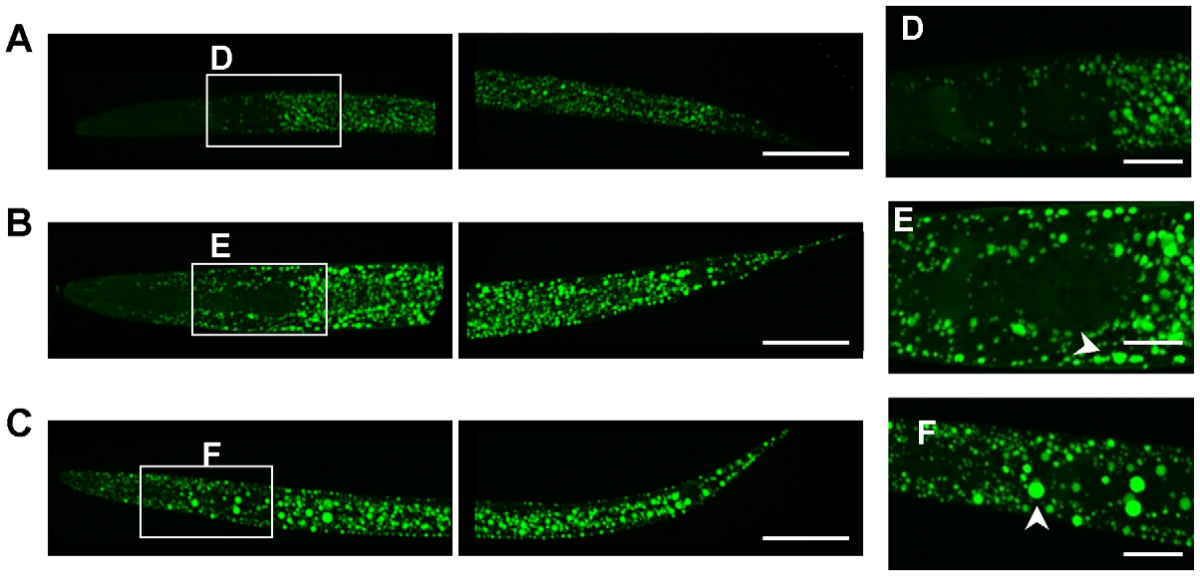
Imaging of lipid droplets in dDR L4 larvae by scanning-laser confocal microscopy. Wild-type animals grown on AL (A), dDR1.5 (B) and dDR0.7 (C) plates were harvested at L4 stage and used for fixative BODIPY 493/503 staining. Images derived from CLS microscopy and are shown as maximum projection of ∼40 images from a z-stack at 0.5 µm interval. The anterior part is on the left, the posterior on the right. (D–F) Detailed view of the pharynx region. Arrow heads indicate enlarged hypodermal LDs. Magnification 630×.

**Figure 7:**
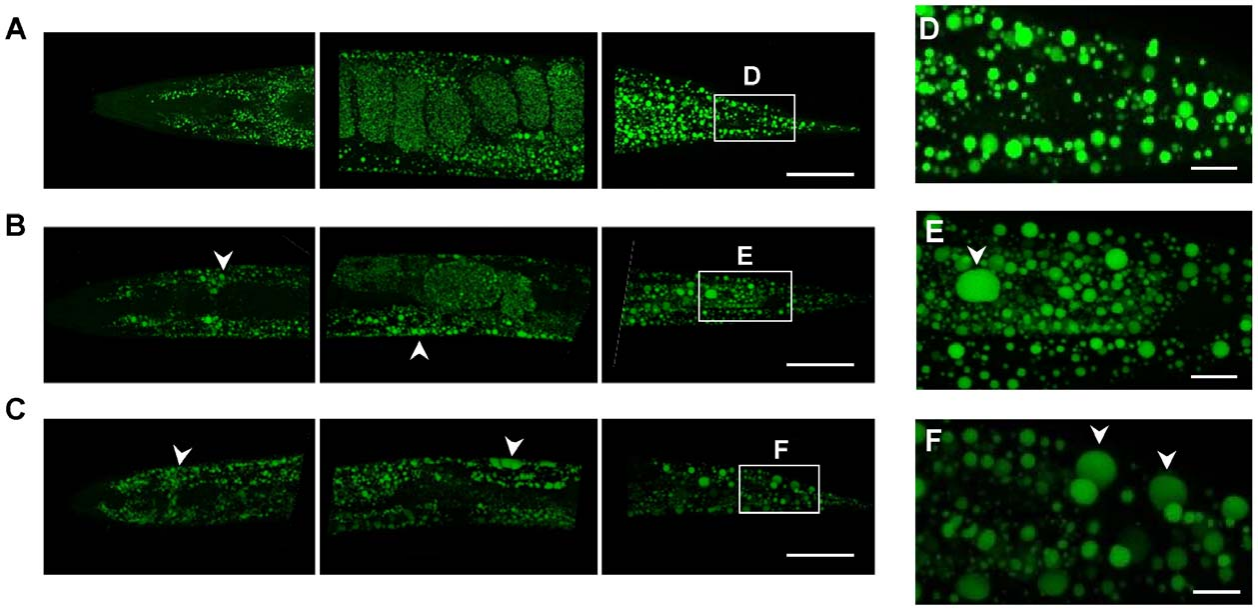
Imaging of lipid droplets in dDR adult worms by scanning-laser confocal microscopy. Fixative BODIPY 493/503 staining of adult wild-type worms cultivated under AL (A), dDR1.5 (B) and dDR0.7 (C) condition. CLS microscopy images of pharynx (on the right), central and tail region (on the left) are shown as maximum projection of 70–110 images from a z-stack at 0.5 µm interval. (D–F) Detailed view of tail region. Arrow heads indicate hypodermal LDs. Magnification 630×.

We next asked whether the increased TAG to protein ratio was associated with changes in the lipid droplet (LD) appearance. Conventional fluorescence microscopy of fixative BODIPY 493/505-stained adult AL fed worms showed a high density of small-sized LDs (**Figure 4**). Interestingly, dDR0.7 and dDR1.5 increased the size of intestinal and hypodermal LDs in worms at the first (**Figure 4**), second (**Figure S3**) and eight (**Figure S4**) day of adulthood. LD expansion under dDR was even visible in corresponding bright-field microscopy images (**Figure 4** **, S3, S4**) and was confirmed by BODIPY 493/505 based vital staining (**Figure S5**) as well as by the Oil red O method (**Figure 4D–F**). Moreover, enlarged LDs were also observed in *eat-2 (ad465)* mutants, a genetic model of DR (**Figure S6**). Control experiments revealed no differences in LD size of adult N2 worms exposed during development to AL plates containing peptone or no peptone (**Figure S7**). To investigate the LD expansion phenotype in more detail, scanning-laser confocal (SLC) microscopy of fixative BODIPY 493/503 stained worms was performed. The enlargement of intestinal and hypodermal LDs under moderate and stringent dDR condition was observed in L2 larvae (**Figure 5**), L4 larvae (**Figure 6**) and adult worms (**Figure 7**). In summary, DR increased the LD size in intestinal and hypodermal cells of larvae and adult worms which were exposed to DR during development.

### dDR increases the relative number of large-sized lipid droplets by a factor of about 2 to 15 depending on the developmental stage

**Figure 8:**
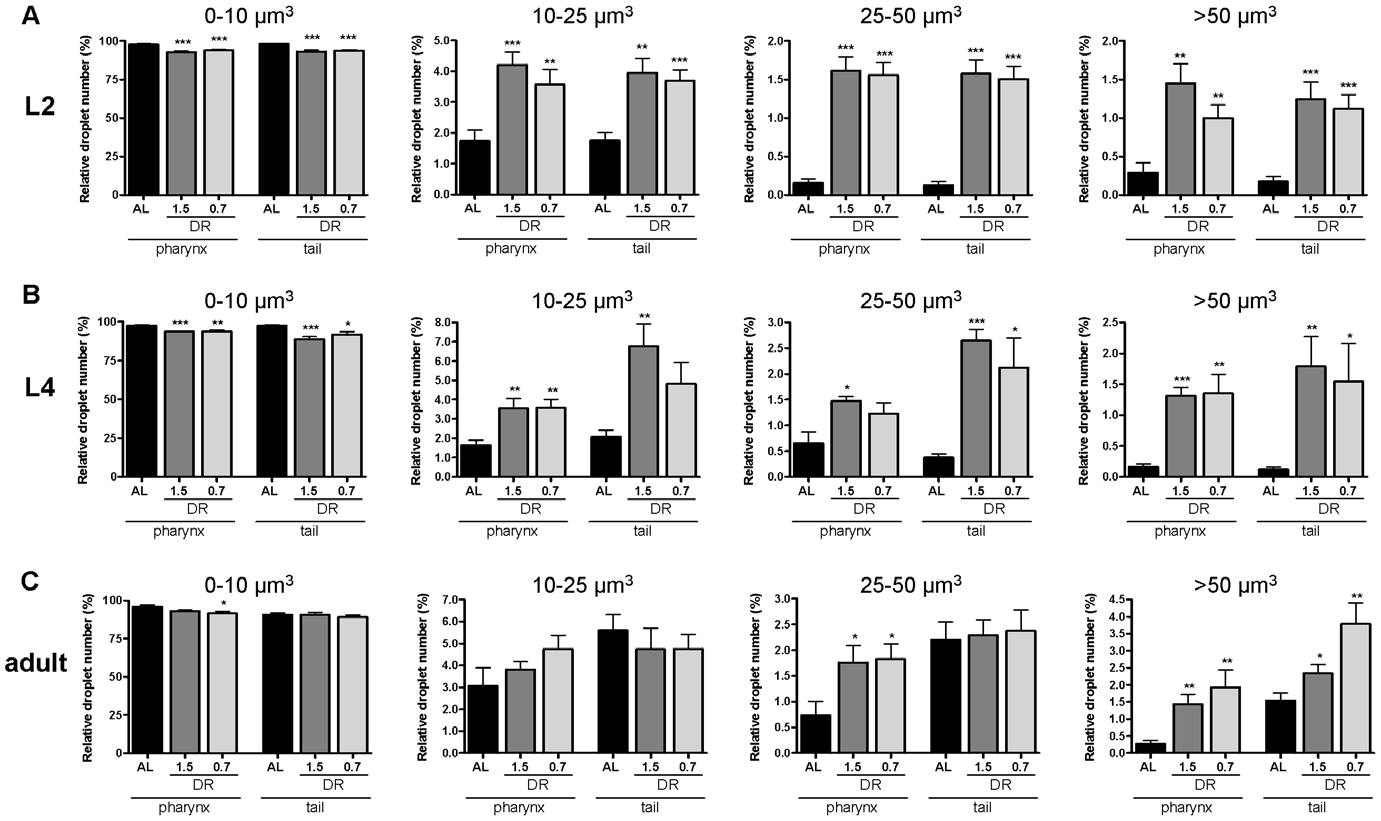
Size classification of BODIPY 493/503-stained lipid droplets in pharynx and tail region of dDR larvae and adult *C. elegans*. The volumes of all BODIPY 493/503-positive droplets (fixative staining) in pharynx and tail region of AL and dDR (dDR1.5, dDR0.7) treated wild-type animals were obtained from single z-stacks of CLS microscopy images. The relative number of lipid droplets (%) that are 0–10 µm^3^, 10–25 µm^3^, 25–50 µm^3^ and >50 µm^3^ in volume was calculated for L2 larvae (A), L4 larvae (B) and adult worms (C). Data derive from eight to ten animals per feeding condition and developmental stage, respectively. Results are shown as mean ± SEM from three independent experiments. (*p<0.05, **p<0.01, ***p<0.001).

To quantify the observed enlargement of LDs in response to dDR, the number of fixative BODIPY-labeled LDs in pharynx and tail regions was calculated from single z-stacks of SLC microscopy images (**Figure 8**). Small-sized LDs<10 µm^3^ in volume represented 88.8 to 97.9% of detected LDs at all developmental stages and at all feeding conditions. In comparison with AL condition, dDR increased the relative number of medium-sized LDs (10–25 µm^3^) of L2 and L4 larvae by a factor of 2.1 to 3.3. The relative abundance of large-sized (25–50 µm^3^) and very large-sized (>50 µm^3^) LDs increased up to 12-fold in dDR L4 larvae compared with AL condition. In tail region of dDR L4 larvae, the relative number of very large LDs was actually up to 15.2-fold higher. The relative number of large-sized LDs (25–50 µm^3^) was up to 2.5-fold increased in pharynx region of dDR adult worms. The percentage of very large LDs (>50 µm^3^) was up to 7.1-fold higher in dDR adult worms. Taken together, the extent of dDR-induced LD expansion was dependent on the respective body region as well as on the developmental stage.

### Under dDR up to 65% of the total LD volume was represented by large-sized LDs (>50 µm^3^)

**Figure 9:**
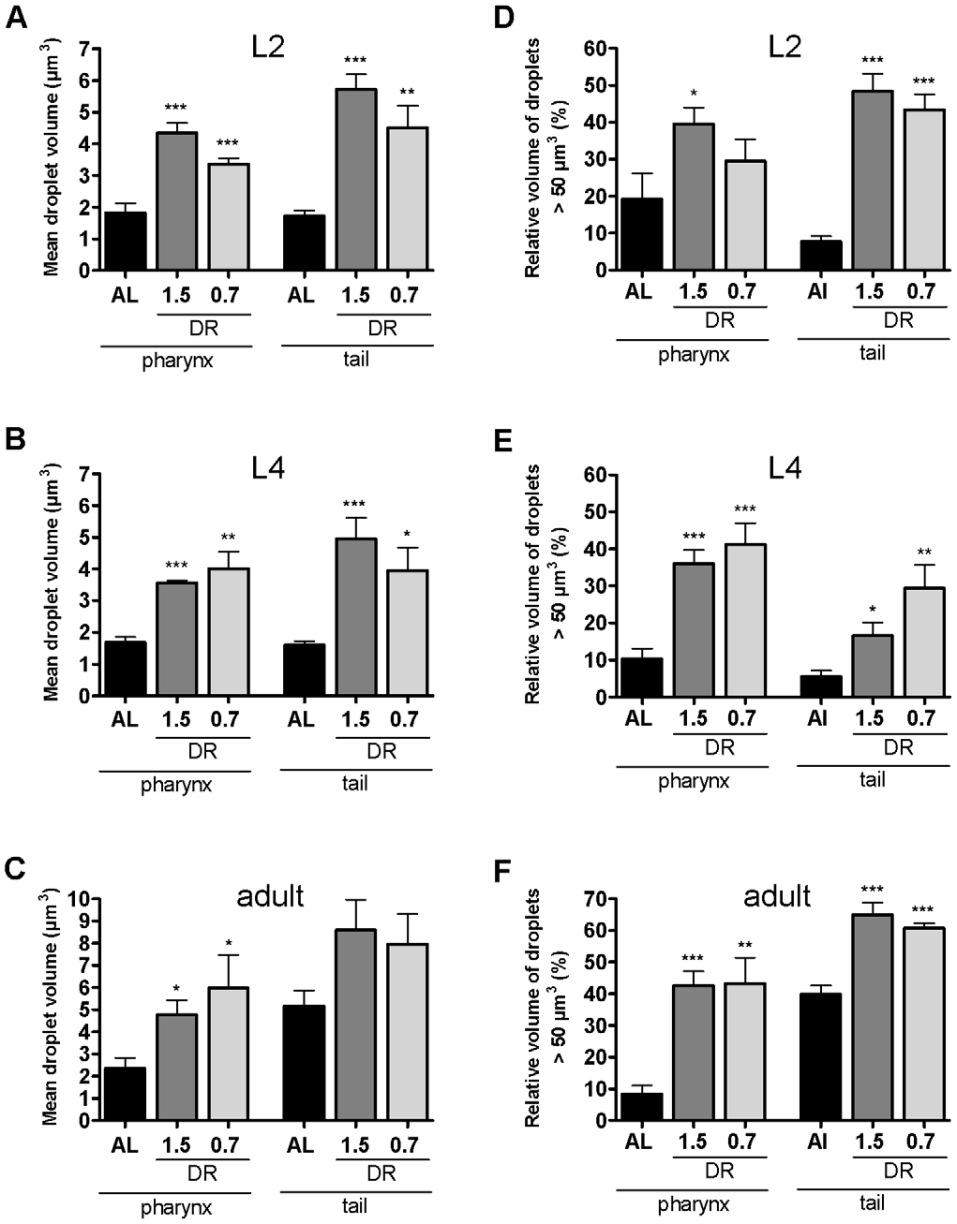
Mean lipid droplet volume and volume-% of large-sized lipid droplets of dDR larvae and adult *C. elegans*. Mean LD volume (µm^3^) (A–C) and percentage volume of large-sized LDs (>50 µm3) on the total volume of all detected droplets (%) (D–F) within pharynx and tail region was quantified in L2 and L4 larvae and adult wild-type worms. Animals were fed on AL or two different dDR conditions (dDR1.5, dDR0.7). LDs were visualized by fixative BODIPY 493/503 staining. LD volumes were calculated from z-stacks of CLS microscopy images. Eight to ten animals were analyzed per condition and experiment. Results are shown as mean ± SEM from three independent experiments (*p<0.05, **p<0.01, ***p<0.001).

The mean LD volume of all BODIPY-labeled LDs in pharynx and tail region was calculated from single z-stacks of CLS microscopy images. Moderate and stringent dDR (dDR1.5, dDR0.7) increased the mean LD volume of L2 larvae, L4 larvae and adult worms in comparison with respective AL condition (**Figure 9A–C**). In pharynx region, the mean droplet size of dDR animals was 1.8 to 2.5-fold increased. Mean droplet volume of tail regions was up to 3.3-fold elevated. As a consequence, the surface to volume ratio of LD was reduced (14–32%) under dDR (**Table S2**). For further comparison, the volume of large-sized LDs (>50 µm^3^) was expressed as percentage of the total LD volume (**Figure 9D–F**). This parameter displays the relative proportion of fat stored in large LDs. In comparison with AL condition, moderate and stringent dDR significantly increased the volume-% of LD fraction >50 µm^3^ by a factor of 1.5 to 6.3 in L2 larvae and 3.0 to 5.3 in L4 larvae, respectively. In adulthood, LD fraction >50 µm^3^ represented 42.6 to 43.3% (AL: 8.37%) and 60.7 to 64.9% (AL: 39.9%) of the total LD volume in pharynx and tail region of dDR fed worms, respectively. Moreover, maximum-sized LDs were enlarged by a factor of about 2 to 5 in dDR animals when compared with AL (**Table S3**). The largest LDs were detected in the tail region of dDR adult worms (dDR1.5: 609±93 dDR0.7: 408±68; mean±SEM of the five largest LDs). Together, in worms subjected to dDR, up to 65% of the total LD volume was represented by large-sized LDs (>50 µm^3^).

### Microarray analysis identifies 124 genes which are consistently responsive to dDR

To identify candidate genes that might be responsible for the dDR induced increase in the TAG to protein ratio and enlargement of LDs we performed gene expression profiling using microarrays. We compared genome wide mRNA stead-state levels of L4 and adult worms which were exposed to AL and dDR (0.7, 1.5) during development. To analyze and interpret gene expression data, we considered all genes that were significantly regulated (fold-change>2.0; p<0.05, t-test) under both dDR conditions relative to AL treated control group (**Table 1**). Genes implicated in lipid metabolism were also selected (**Table S4**).

**Table 1:** Summary of dDR response genes in C. elegans.

		Fold change of regulation^b^			
		L4		adult	
Gene	Description^a^	DR1.5	DR0.7	DR1.5	DR0.7
**Fatty acid metabilism**					
*fat-5*	Δ-9 fatty acid desaturase	3.7	3.6	2.3	2.5
Y48A6B.9	putative mitochondrial trans-2-enol-CoA reductase (FA elongation)^c^	2.7	3.9	2.7	2.7
Y53G8B.2	diacylglycerol acyltransferase (DGAT)	3.5	5.1	2.6	2.6
*acs-2*	Fatty acid acyl-CoA synthetase	2.4	3.2	8.3	12.0
*acs-7*	Fatty acid acyl-CoA synthetase	−4.5	−2.3	−5.3	−7.6
T20B3.1	Carnitine acyltransferase	2.4	3.7	10.1	9.2
K09H11.1.1	Acyl-CoA dehydrogenase, mitochondrial	2.2	2.6	2.0	2.2
F58F9.7.1	Acyl-CoA oxidase, peroxisomal	2.1	2.7	2.4	2.3
F58F9.7.3	Acyl-CoA oxidase, peroxisomal	2.0	2.7	2.6	2.4
*lips-6*	Triacylglycerol lipase (class 2)	11.1	12.8	2.7	4.0
C40H1.8.1	Predicted lipase (class3)	−3.7	−4.2	−4.0	−3.0
*lips-11*	Triacylglycerol lipase	−4.3	−5.6	−3.1	−4.3
*lips-12*	Triacylglycerol lipase (class2)	−2.2	−2.3	−3.3	−3.9
*lipl-5*	Triglyceride lipase- cholesterol esterase	−3.7	−4.2	−2.4	−2.2
**Lipid transport/storage**					
*far-3*	Fatty Acid/retinol binding protein	10.7	13.4	3.7	3.1
F22E5.1	Lipid storage	−2.1	−4.3	−20.7	−17.9
*swt-1*	Sweet sugar transporter family member; lipid storage	2.1	2.3	4.0	4.0
*vit-1*	Lipoprotein, lipid transporter activity	−2.5	−6.2	−15.9	−49.5
**Other metabolic pathways**					
*ugt-63*	UDP-glucuronosyl and UDP-glucosyl transferase	−6.6	−9.5	−29.1	−30.5
*ugt-15*	UDP-glucuronosyl and UDP-glucosyl transferase	42.7	31.5	6.3	6.8
*ugt-8*	UDP-glucuronosyl and UDP-glucosyl transferase	−2.6	−2.6	−7.2	−5.8
*ugt-53*	UDP-glucuronosyl and UDP-glucosyl transferase	−3.0	−4.0	−3.0	−3.7
*ugt-18*	UDP-glucuronosyl and UDP-glucosyl transferase	12.2	13.9	34.0	41.7
Y4C6B.6	Beta-glucocerebrosidase; lysosome organization, carbohydrate and sphingolipid metabolic process	6.2	7.0	31.8	34.1
*dhs-18*	Dehydrogenase, short chain	2.2	3.5	3.8	3.7
*dhs-9* (Y32H12A.3.1)	Short-chain dehydrogenase/reductase	2.2	3.2	2.3	2.6
*dhs-9* (Y32H12A.3.2)	Short-chain dehydrogenase/reductase	2.3	3.2	2.2	2.5
*cth-1* (F22B8.6.1)	Putative cystathionine gamma-lyase, amino acid metabolic process	3.3	2.6	2.1	2.1
*cth-1* (F22B8.6.2)	Putative cystathionine gamma-lyase, amino acid metabolic process	3.3	2.6	2.1	2.2
*asns-2*	Asparagine synthase (glutamine-hydrolyzing)	2.2	3.6	7.8	7.6
C01B10.7	Transferase activity	−2.3	−4.5	−5.3	−7.6
C42D4.2	Carboxylesterase and related proteins	−2.0	−4.1	−8.2	−10.9
F10C2.3	catalytic activity	2.6	2.9	3.0	2.5
F54F3.4	Reductase with broad range of substrate specificities	2.9	4.1	5.0	5.7
**Regulation of lifespan**					
*dct-8*	DAF-16/FOFO controlled, germline Tumor affecting	8.1	26.4	8.3	9.8
*dod-23*	Downstream of DAF-16 (regulated by DAF-16) family member	−2.4	−2.9	−2.2	−2.6
*hsp-12.3*	Small heat-shock protein, response to heat	4.9	6.3	3.0	3.0
*mtl-2*	Metallothionein, functions in metall detoxification and homeostasis and stress adaptation; plays a role in regulating growth and fertility, determination of adult lifespan	3.2	3.9	2.8	3.1
T16G1.7	Orthologous to human gene ALIAS DLC1 CANDIDATE TUMOR SUPPRESSOR GENE (DLEC1)	8.4	10.5	10.2	13.6
**Regulation of transcription**					
*djr-1.2*	DJ-1 (mammalian transcriptional regulator) related	3.2	4.7	2.2	3.0
*nhr-74*	Nuclear hormone receptor	2.8	2.7	−16.5	−18.4
*nhr-117* (F16B4.12a)	Nuclear hormone receptor	2.3	2.8	4.1	4.8
*nhr-117* (F16B4.12b)	Nuclear hormone receptor	2.3	2.8	4.1	4.8
*nhr-244*	Nuclear hormone receptor	2.4	2.6	−10.2	−9.4
*oac-20*	O-Acyltransferase homolog	−2.1	−2.5	−2.6	−4.8
**Immune response**					
*clec-169*	C-type lectin	−2.7	−3.8	−6.5	−4.9
*clec-68*	C-type lectin	6.8	5.4	2.3	4.1
*clec-150*	C-type lectin	−2.2	−2.7	−3.6	−4.5
*clec-50*	C-type lectin	−2.5	−2.5	−3.0	−3.6
*clec-237*	C-type lectin	−3.6	−4.0	−2.9	−3.5
*clec-4*	C-type lectin	2.2	2.6	−2.2	−2.4
*clec-97*	C-type lectin	−2.7	−3.1	−3.3	−2.8
F35E12.5	CUB-like domain bearing protein	−3.0	−3.8	−24.7	−30.3
F55G11.4	CUB-like domain bearing protein	−3.7	−3.9	−11.5	−11.8
F55G11.7.2	CUB-like domain bearing protein	−2.0	−2.2	−5.8	−6.7
*ilys-5*	Lysozyme activity	−2.4	−2.3	−18.6	−25.8
*spp-17*	Saposin-like family member; Saposin B	−2.0	−3.3	−7.2	−14.6
**Detoxification and Defense**					
*cyp-34A1*	Cytochrome P450 CYP2 subfamily member	9.2	16.4	3.8	4.0
*cyp-35C1*	Cytochrome P450 CYP2 subfamily member	−2.9	−3.5	−16.6	−20.5
*cyp-35D1*	Cytochrome P450 CYP2 subfamily member	−5.5	−8.1	−15.7	−33.0
*cyp-35A5*	Cytochrome P450 CYP2 subfamily member, lipid storage	−4.3	−6.5	−22.0	−27.7
*cyp-35A3*	Cytochrome P450 CYP2 subfamily member, lipid storage	−2.8	−3.3	−12.5	−13.0
*scl-6*	Defense-related protein containing SCP domain	−3.7	−2.9	−53.2	−46.7
**Transport**					
*amt-4*	Ammonia permease	−2.1	−2.8	−7.6	−7.3
C18D1.2	Permease of the major facilitator superfamily	4.5	6.4	3.9	5.5
F56A4.10	Permease of the major facilitator superfamily	−2.3	−2.4	−6.2	−8.7
Y19D10A.8	Transmembrane transport, predicted	−2.1	−2.2	−3.1	−9.5
**Other functions**					
C15C8.3	Aspartyl protease	−2.1	−3.7	−24.3	−14.8
*ins-12*	Hormone activity	2.5	2.5	−3.4	−4.3
*scav-5*	Plasma membrane glycoprotein CD36 and related membrane receptors; cell adhesion	−2.1	−2.6	−6.2	−6.2
*str-9*	Seven TM Receptor; 7-transmembrane olfactory family member	2.5	3.1	2.3	2.9
*wrt-8*	Sonic hedgehog and related proteins; cell-cell signaling	2.3	2.7	6.4	8.0

The table shows the predicted molecular functions and fold changes in gene expression of DR response genes that were commonly regulated under moderate and stringent DR (DR1.5 and DR0.7) in L4 and adult wild-type worms. Genes with unknown function are excluded in this table. Criteria for inclusion in this data set were a fold-change >2.0 and significance threshold of p-value<0.05.

a Description of genes is based on the gene ontology (GO) annotation for *C. elegans* (WormBase, www.wormbase.org., release WS 198) unless otherwise noted.

b Fold changes are understood between the DR and AL group. A positive number indicates a higher gene expression in DR animals. In case of down-regulated genes, the fold-change was calculated as 1/ratio and a minus was added to the quotient. A negative number consequently indicates a lower gene expression in DR animals.

C Description is based on Kyoto Encyclopedia of Genes and Genomes (KEGG, www.genome.jp/kegg/, release 61.0).

**Figure 10:**
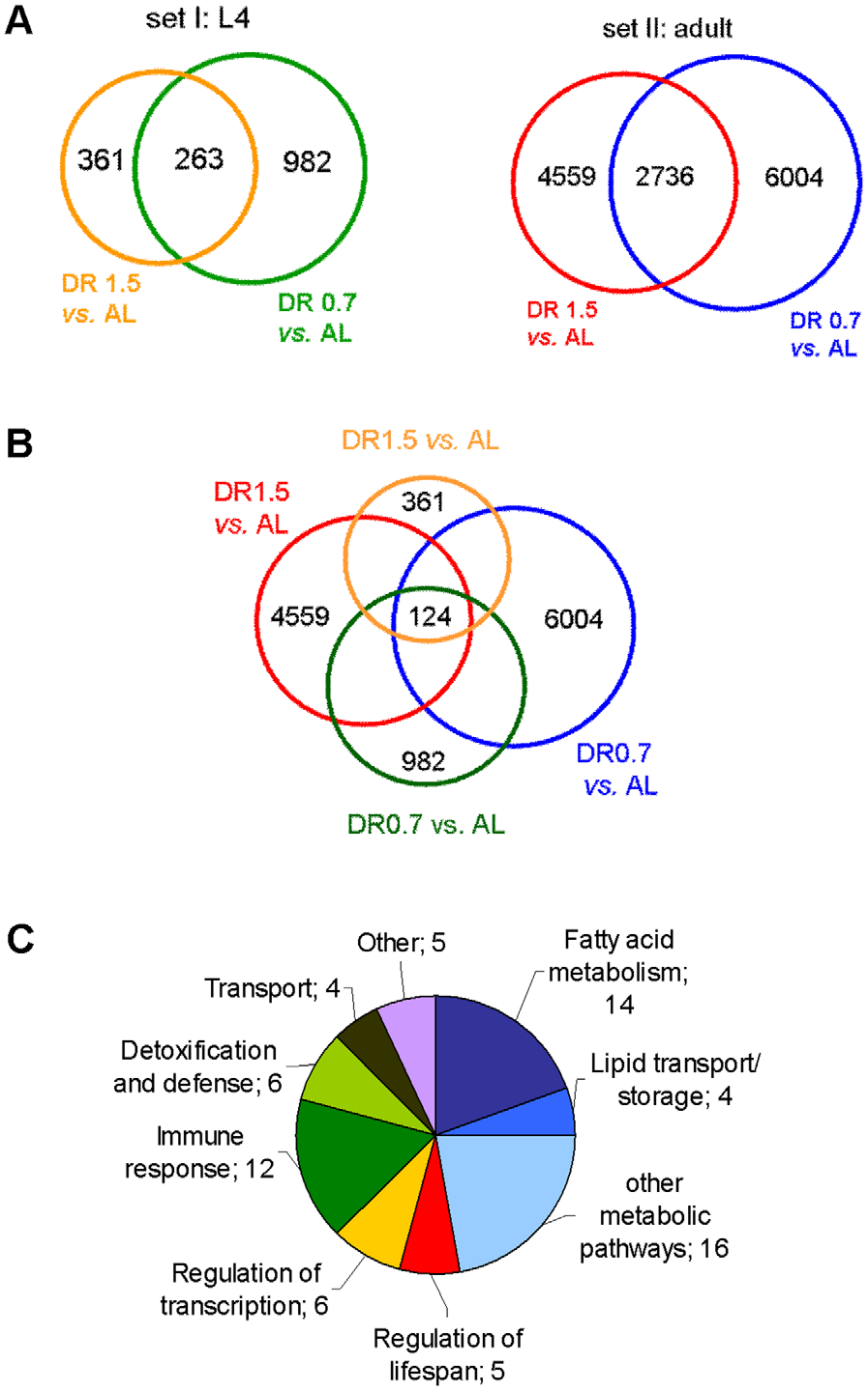
Illustration of shared genes regulated under stringent and moderate dDR in wild-type L4 larvae and adults. (A) Comparison of significantly up- and down-regulated genes in L4 larvae (set I) and adult worms (set II) grown under dDR1.5 and dDR0.7 relative to AL treated control group. Numbers of regulated genes within each subset are listed. Intersections represent shared regulated genes. Selected criteria for inclusion in the gene subsets were a fold change in expression to exceed 2-fold and a confidence level of 95% (p<0.05, t-test). (B) Combination of the 2 sets revealed shared regulated genes (‘dDR response genes’) used for subsequent analysis. (C) The pie chat represents the functional categories of shared dDR response genes based on their related molecular function using gene ontology (GO) annotation for *C. elegans* (WormBase, release WS 198). Genes with unknown function are excluded in this analysis.

We found 263 genes that were significantly up-regulated or down-regulated in L4 larvae cultivated under dDR1.5 and dDR0.7 (**Set I,** **Figure 10**). At the adult stage, 2736 genes were significantly regulated under dDR0.7 and dDR1.5 (**set II,** **Figure 10A**). The combination of these two gene sets identified 124 shared genes that were significantly regulated at both developmental stages and both dDR conditions (**Figure 10B**). These genes were considered as dDR response genes. Examination of the predicted molecular functions using the gene ontology annotation for *C. elegans* revealed assignments for 72 of the 124 dDR response genes (**Table 1**). Related annotations were combined into broader categories including fatty acid metabolism and other metabolic processes, lipid transport and storage, regulation of lifespan, regulation of transcription, immune response, detoxification and defense, transport and other functions (**Figure 10C**).

### dDR regulates genes of the fatty acid metabolism and other metabolic pathways

14 of the 72 annotated genes regulated in response to dDR are predicted to encode enzymes involved in fatty acid metabolism (**Table 1**, see also **table S4**). We identified several genes of mitochondrial and peroxisomal ß-oxidation up-regulated under dDR in L4 larvae and adults. These include the mitochondrial acyl-CoA synthetase encoding gene *acs-2*, one carnitine acyltransferase (T20B3.1), an acyl-CoA dehydrogenase (K09H11.11) and two splice variants of a putative acyl-CoA oxidase (F58F9.7). Four genes encoding triacylglycerol lipases were down-regulated in response to dDR. One lipase encoding gene (*lips-6*) was up-regulated and showed increased fold changes at L4 compared to the adult stage.

The diacylglycerol acyltransferase (DGAT) gene (Y53G8B.2) was significantly up-regulated in dDR L4 larvae and adults. DGAT is a key enzyme of *de novo* synthesis of triacylglycerols (TAG). Expression levels of *fat-5* encoding a Δ9 desaturase were significantly up-regulated under dDR relative to AL condition. Further, expression of Y48A6B.9 gene was stimulated in response to dDR. Y48A6B.9 is predicted to encode a putative trans-2-enol-CoA reductase participating in fatty acid elongation. Fatty acid/retinol binding protein encoding *far-3* was up-regulated and exhibited increased fold changes at L4 in comparison to the adult stage. Two other dDR response genes, F22E5.1 and K02D7.5 (*swt-1*), are predicted to function in lipid storage. A strong down-regulation (up to 49.5 fold in dDR0.7 fed adults) was observed for *vit-1*, which encodes a lipoprotein functioning in lipid transport. We also found that dDR altered the expression of genes involved in other metabolic pathways. For example, of five UDP-glucuronosyl/UDP-glucosyl transferase encoding genes expected to function in carbohydrate metabolism and lipid glycosylation, three genes were repressed, whereas two genes were up-regulated. Further, the expression of two genes predicted to function in amino acid metabolism, one putative cystathionine gamma-lyase (*cth-1*) and one asparagine synthase (*asns-2*), was up-regulated under stringent and moderate dDR in L4 larvae and adult animals.

### dDR regulates genes involved in life span extension, stress response and transcription

As expected, dDR altered the expression of genes predicted to function in the determination of lifespan (**Table 1**). Furthermore, we identified several dDR responsive genes involved in immune response (**Table 1**): seven C-type lectin encoding genes, three genes encoding a CUB-like bearing protein, saposin B encoding *spp-17* and *ilys-5*, which encodes an invertebrate lysozyme family member. Interestingly, expression of these genes was consistently down-regulated under dDR with exception of C-type lectin encoding gene *clec-68*, which was up-regulated during the L4 and adult stage. dDR also influenced the expression of six genes related to detoxification and defense, including cytochrome P450 family members and *scl-6*, which encodes a defense-related protein. Of these genes, five were down-regulated, whereas only *cyp-34A1* was up-regulated under dDR. Another group of dDR-response genes is involved in the transcriptional regulation. We found that three nuclear hormone receptor (NHR) encoding genes (*nhr*-74, *nhr-117*, *nhr-244*) were consistently up-regulated in dDR L4 larvae (Table 1). In contrast, dDR led to the repression of *nhr-74* and *nhr-244* in the adult stage. Further, expression of *djr-1.2* encoding a DJ-1 (mammalian transcriptional regulator) related protein was stimulated in response to dDR, whereas O-acyltransferase homolog *oac-20*, an, was down-regulated.

## Discussion

In comparison to other DR protocols on solid medium [7], [24], [32] our method was established to study effects of DR during development. This approach was named “development-DR (dDR)”. It offers several advantages. First, our method minimizes possible side-effects of antibiotic or heat treatment due to the fact that exclusion of peptone and serial dilution of living *E. coli* is the sole intervention. Second, we were able to control the food availability per worm using flow-cytometry based sorting of a defined number of embryos per plate. We show that serial dilution of the food source results in a dose-dependent reduction of body size indicating standardized dDR conditions. Third, our method enables the application of DR during development from hatching throughout development. Several other regimes, including diluting bacteria in liquid medium, axenic media, dietary deprivation or DR on agar plates, initiate DR in *C. elegans* at L4 stage or during adulthood to avoid detrimental effects on development or surviving [7], [11], [14], [26]–[29], [32], [35]. Using our method, the onset of DR from hatching throughout larval development did not induced larval arrest, dauer stage or and an adult reproductive diapause [33]. However, developmental time was slightly delayed under dDR (first egg lay: AL: ∼72 h, dDR1.5: ∼74 h; dDR0.7: ∼76 h). In summary, the use of living bacteria, the controlled application of the extent of dDR and the applicability during development are the main advantages of our dDR protocol. Of course, an influence of peptone or metabolic changes of living bacteria growing on AL and DR plates cannot be excluded. In control experiments using AL plates containing a thick bacterial lawn we observed not influence of peptone on LD size.

A reduction in body size under nutrient-limiting conditions has been observed in several species including *D. melanogaster* and mice [36], [37]. In line with this, we found an inverse linear relationship between body size and the extent of dDR in adult worms exposed to DR during development. The observed body sizes of AL and dDR treated worms were similar to those reported in other studies performing DR in *C. elegans* [25], [38].

The dDR induced reduction in body size was accompanied by a reduced protein content of adult worms. A close correlation between body size and protein content in *C. elegans* was also observed by using different *E. coli* strains [39]. It has been proposed that body size is controlled by the insulin/IGF-1 signaling pathway, which responds to the nutritional state and promotes cell growth by increasing the protein synthesis. DR-dependent (plate, killed bacteria) reduction of body size in adult *C. elegans* is mediated by the sensory EGL-4/SMA/MAB pathway which regulates hypodermal endoreduplication [25]. The reduction in body size during dDR might also arise from these pathways and/or might be linked or accompanied by a decreased synthesis of phospholipids. In *C. elegans*, the glycerophospholipids phoshoethanolamine (PE) and phosphatidylcholine (PC) account for 55% and 32% of the total phospholipids, respectively [40]. Both, *de novo* synthesis of phospholipids and triglycerides (TAGs) require diacylglycerol (DAG) as substrate. Conversion of DAG into TAG is catalyzed by diacylglycerol transferase (DGAT) [41]. Gene expression analysis revealed that moderate as well as stringent dDR led to an up-regulation of the DGAT encoding gene Y53G8B.2. Assuming an increased *de novo* TAG synthesis by DGAT, this might cause substrate deficiency for the synthesis of phospholipids which are the major component of cell membranes. Thus, dDR-dependent reduction in body size might be mediated by a reduced polyploidy, synthesis of protein and/or phospholipid *de novo* synthesis.

In *C. elegans*, fat is mainly stored in LDs of the intestine and hypodermis [42], [43]. Surprisingly, moderate as well as stringent DR during development led to a remarkable LD expansion in intestinal and hypodermal cells. The LD expansion was found in L2 larvae, L4 larvae and adult worms which were subjected to DR during development. Of note, the observed LD phenotype under dDR is considerable distinct from the phenotype of fasted animals. Upon fasting, L4 larvae and adult worms mobilize TAGs from intestinal fat stores, which causes a drastic decrease in the number and size of LDs [14], [21], [44], [45].

Gene expression analyses identified several lipogenesis genes that were up-regulated under moderate (dDR 0.7) and stringent dDR (dDR 1.5) in L4 larvae and adult worms. These genes are involved in lipid synthesis (i. e. Y53G8B.2/DGAT), lipid binding (i. e. *far-3*) and fatty acid desaturation (i.e. *fat-5*/Δ9 fatty acid desaturase). Assuming that gene expression is translated into enzymatic activity, up-regulation of these genes might increase *de novo* synthesis of TAGs. This hypothesis is supported by our finding that dDR led to an increased TAG to protein ratio in L4 larvae and adult worms. In line with this, mild calorie restriction in mice led to fat accumulation [46].

Studies indicate that storage efficiency of TAGs into LDs is likely dependent on the generation of monounsaturated fatty acids (MUFAs) [47], [48]. For example, it has been demonstrated in chinese hamster ovary cells and in *Drosophila* that supplementation with oleic acid (C18:1n9) leads to TAG accumulation [49], [50]. Further, supplementation with vaccenic acid (C18:1n7) increased TAG levels and LD size in *C. elegans* peroxisomal ß-oxidation mutants [43]. Here, we found an up-regulation of the Δ9 desaturase encoding gene *fat-5*. FAT-5 catalyzes the desaturation of palmitic acid (16∶0) into palmitoleic acid (C16:1), which serves as substrate for elongation to vaccenic acid [51], [52]. Thus, increased *fat-5* expression may lead to enhanced vaccenic acid levels, which might promote TAG accumulation and enlargement of LDs under dDR. The DGAT encoding gene, which is up-regulated under dDR, also links TAG synthesis to LD size. It has been shown, that mammalian DGAT translocates to the LD surface when fatty acid enter the cells in order to promote lipid production and storage [53]. Taken together, dDR might enlarge LDs via up-regulation of lipogenesis genes such as *fat-5* and DGAT.

The ratio of surface phospholipids to core neutral lipids might be an important determinant of LD size. Depletion of phosphatidylcholine (PC), which represents the most abundant phospholipid in the LD monolayer, leads to enlargement of LDs ([54]–[56]. PC can be synthesized either from choline by the Kennedy pathway or by S-adenosylmethionine (SAM) dependent methylation of phosphatidylethanolamine in mammals or of phosphoethanolamine in nematodes and plants [57]–[59]. Knockdown of the key enzyme of the Kennedy pathway, CTP:phosphocholine cytidylyltransferase (CCT), leads to decreased PC levels and to an drastically increased LD size in *Drosophila* S2 cells [49], [55]. Likewise, repression of genes (*sams-1*, *pmt-1*) mediating methylation-dependent PC synthesis in *C. elegans* leads to large intestinal LDs [56], [60]. More recently, SREBP-1 has been identified as a transcriptional activator of genes involved in the one-carbon cycle and LD accumulation [60]. SREBP-1 target genes include *sams-1*, *pmt-2*, folic acid transporter *folt-2* and several other genes. Because key genes of the PC synthesis are not consistently regulated under dDR in larvae and adult worms, dDR induced enlargement of LD seems to be not mediated by a perturbed SAM-dependent PC synthesis.

It has been shown [56] that LD expansion in *sams-1* and *pmt-1* depleted *C. elegans* was associated with reduced PC levels and increased TAG content indicating a reciprocal regulation of PC and TAG synthesis. Thus, we speculate that up-regulation of DGAT under dDR may indirectly reduce *de novo* synthesis of phospholipids which leads to the LD enlargement under dDR. One model explaining expansion of LDs under conditions of reciprocal regulation of TAGs and phospholipids involves the fusion of LDs [49], [55], [61]. It has been reported that during incorporation of lipids into LDs, PC homeostasis at the expanding monolayer is essential to stabilize the organelles and to prevent their coalescence [55], [62]. In turn, PC deficiency during LD growth leads to fusion of LDs because of instability [55], [62]. We therefore speculate that LD expansion by fusion could be a dDR-induced mechanism to reduce the surface to volume ratio of LD, which might protect stored TAGs against lipolysis.

In *C. elegans*, only a small number of genes modulating LD size were identified. It has been shown, that mutations in genes encoding enzymes of the MAOC-1/DHS-28/DAF-22 peroxisomal ß-oxidation pathway cause LD expansion in *C. elegans* intestinal cells [43], [63]. Functional loss of ACS-3/acyl-CoA synthetase resulted in the formation of large intestinal LDs due to an elevated fatty acid uptake and increased *de novo* lipid synthesis [42]. LD expansion has been also reported for *C. elegans* deficient for the KLF-3/Krüppel-like transcription factor [64]. Moreover, it has been shown that deletion of *lbp-5* encoding a lipid-binding protein (LBP) leads to enlarged intestinal LDs in the worm [65]. Since all of these known *C. elegans* genes modulating LD size are not consistently regulated by dDR in larvae and adult worms we suggested that they play no substantial role for the observed enlargement of LDs under dDR. However, we cannot exclude a post-transcriptional effect of dDR on these genes.

LD expansion in *C. elegans* mutants were observed in the intestine but not in other fat storage sites [42], [43], [45], [64], [65]. In the present study, we found that dDR causes an expansion of LDs in the intestine as well as in the hypodermis. The formation of enlarged LDs has been functionally associated with an increased resistance of stored lipids to lipolysis. Studies indicate that due to the reduction of the LD surface relative to its volume, lipids are less accessible to membrane associated lipases [43], [49]. In *Drosophila*, large LDs were slower metabolized than smaller ones and provided a survival advantage during starvation [43]. In *C. elegans*, peroxisomal ß-oxidation *dhs-28* mutant exhibited enlarged LDs in the intestine that were resistant to fasting and lipase induced lipolysis [43], [63]. Over-expression of adipose triglyceride lipase *atgl-1* (C05D11.7) caused a drastic reduction of TAG levels in wild-type but to a much lesser extent in *dhs-28* animals. We observed a reduction of the surface to volume ratio of LD under dDR in comparison to AL condition. Moreover, of dDR response genes four out of five lipase genes were down-regulated (*lipl-5*, *lips-6*, *lips-12* and C40H1.8), which may indicate decreased lipolysis rates under dDR. Thus, we assume that expansion of LDs in the one case and reduced activity of lipases in the other case might be a protective mechanism to prevent excessive breakdown of stored lipids and to release energy stores more slowly during dDR.

In conclusion, using an optimized DR protocol we demonstrated that DR subjected to *C. elegans* during development increases the triglyceride to protein ratio, enlarges lipid droplets and alters the expression of genes functioning in lipid metabolism. These changes might be an effective adaptation to conserve fat stores in animals which were exposed to dietary restriction during development.

## Materials and Methods

### Strains and Maintenance

Wild-type *C. elegans* Bristol N2 and mutant strains *daf-2(e1370)* and *eat-2(ad465)* were used. Nematodes were cultivated at 20°C on Nematode Growth Medium (NGM) agar plates seeded with *Escherichia coli* OP50 as food source [66]. Strains were obtained from the *Caenorhabditis* Genetics Center (Minnesota, USA).

### Dietary restriction on agar plates

To induce dDR on agar plates bactopeptone (BD, Heidelberg, Germany) was omitted from the standard NGM recipe [32], [66]. *E. coli* OP50 suspension was cultivated in DYT medium at 37°C until reaching an optical density (OD_600 nm_/ml) of 1.5. A serial dilution of *E. coli* OP50 suspended in M9 buffer was prepared ranging from OD_600_ = 6.0 to OD_600_ = 0.3. For dDR conditions, 250 µl of the respective *E. coli* suspension was seeded onto the dDR plates. For *ad libitum* (AL) condition, standard NGM agar seeded with *E. coli* OP50, OD_600_ = 1.5 was used. Plates were incubated at 37°C for 16 h. For all experiments, nematodes were synchronized by hypochlorite treatment of gravid adults. To standardize food availability, 500 eggs per plate were sorted via a cytometry-based object parametric analysis and sorting system (COPAS Biosort, Union Biometra, Geel, Belgium) and cultivated at 20 C until reaching L2, L4 or adult stage. Animals were transferred daily onto fresh plates at L4 and adult stages to prevent starvation.

### Preparation of worm homogenates and biochemical measurements

Wild-type nematodes were cultivated under AL and two different dDR conditions (dDR0.7, dDR1.5) until reaching the L4 and adult stage, respectively. For each replicate, 2000 to 2500 L4 larvae and 800 to 1000 adult worms were collected via the COPAS Biosort system. Animals were homogenized in 100 µl buffer (150 mM NaCl; 1 mM EDTA; 50 mM Tris-HCl, pH 7.5; 0.5% CHAPS) using the Precellys 24 homogenizer (Peqlab, Erlangen, Germany) and ceramic beads (1.4 mm diameter). Cell debris was removed by centrifugation at 21,000 *g* for 20 minutes. Homogenates were used in 96-well format for colorimetric determination of the triacylglycerol (TAG) and protein content as well as for quantification of auto fluorescent ‘age pigment’. The triacylglycerol (TAG) content was determined using an enzymatic assay (Analyticon diagnostics, Lichtenfels, Germany) and a TAG standard (Biovision, Hannover, Germany) according to the manufacturer's directions. The protein content was measured using the Pierce® BCA protein assay kit (Thermo Fisher Scientific, Bonn, Germany) and bovine serum albumin (BSA) as protein standard.

### Thin layer chromatography

Worms collected with COPAS Biosort were analysed by thin layer chromatography [67] after thawing from −80°C. Lipids were extracted from 900 homogenised worms (*Precellys24*; full speed; 2×5 sec.) with 375 µl methanol, 1250 µl methyl-tert-buthyl-ether [63], and 312 µl H2O (3∶10∶2.5 [v]) and solved in 30 µl chlorophorm. The probes were applied on *Polygram SIL G* pre-coated TLC sheets (20×20 cm; 0.2 mm silicagel; Macherey-Nagel) using hexane∶diethyl-ether∶formic acid (20∶20∶1 [v]) as mobile phase. Lipid spots were stained [68] for 20 seconds in a dip solution containing 10% copper(II) sulphate, 8% phosphor acid and 5% methanol. The quantification was done by calculating the spot intensity per area [pixel/µm2] using *AlphaEaseFC* (Biorad). Trioleine (Sigma Aldrich, Germany) and a phospholipid mixture (Sigma Aldrich, P3817) were used as standards. All results are presented as mean values between the normal and diluted probes.

### Determination of the body proportion

Gravid adult wild-type worms cultivated under AL and different dDR conditions (dDR0.3 to dDR6.0) were harvested and anesthetized in 2% NaN_3_. Bright-field microscopy images were obtained using a Zeiss Axio Observer.D1 inverted microscope equipped with an Axiocam MRm camera (Zeiss, Jena, Germany). Magnification of animals was 50-fold (5×/0.12 objective). Length (µm), width (µm), perimeter (µm) and area (µm^2^) of single worms was obtained using the AxioVision software (Release 4.8, Zeiss, Jena, Germany). The body volume (nl) was calculated using an adapted cylinder volume formula which includes the worm's area and perimeter [69], [70]. In addition, time of flight (TOF; arbitrary unit, AU) and extinction (Ext, AU) of single nematodes was measured via a COPAS Biosort. TOF and Ext values are approximate values for the axial length and volume of the worms, respectively [71]. Using flow cytometry, 500 to 2,000 animals were analyzed for each condition in three independent experiments.

### Motility and pharyngeal pumping rate

For analysis of animal motility and pharyngeal pumping rate, 10 animals per agar plate were cultivated under AL and two different dDR conditions (dDR0.7, dDR1.5), respectively. Single nematodes were analyzed at the first day of adulthood using a Zeiss SteREO Discovery V8 binocular microscope (Zeiss, Jena, Germany). For analysis of animal motility, single worms were recorded for 20 seconds at 8.0-fold magnification using a Zeiss AxioCam ICc 1 and Zeiss AxioVision software (Release 4.8). A worm tracking software (WormTracker 2.0.25, Thomas Bornhaupt, Neustadt adW., Germany) was used to calculate the body bending frequency (Hz), whole animal motility (mm s^−1^) and head motility (mm s^−1^) [72]. For measurements of pharyngeal pumping rate, the pharynx was filmed for 40–60 seconds at 63-fold magnification using a Canon camera (Legria HF20). The pharyngeal pumping rate (pumps/min) was counted on slowed films. 20 randomly chosen animals were recorded for each condition in at least three independent experiments.

### Oil Red O staining

AL fed and dDR (dDR0.7, dDR1.5) wild-type worms were harvested at day one of adulthood. Animals were washed in PBS buffer and fixed with 4% paraformaldehyde (PFA) for 15 min. After three freeze and thaw cycles in liquid nitrogen, worms were washed in PBS buffer, followed by a dehydration step in 60% isopropanol. Animals were stained in filtered Oil Red O staining solution (60% Oil Red O stock solution (5 mg/ml isopropanol)/40% distilled water) over night. After washing in PBS, worms were analyzed using an Axio Observer D1 inverted microscope (Zeiss, Germany). Photographs of 10–15 worms per feeding condition were taken by AxioCam MRm camera (Zeiss, Germany) and a 20×/0.50 M27 objective at a fixed exposure time. All experiments were performed three times.

### BODIPY™ 493/503 staining

Synchronized wild-type worms grown under either AL or dDR condition (dDR0.7, dDR1.5) were harvested at different developmental stages (L2, L4, adulthood). Fixative and vital BODIPY™ 493/503 (Invitrogen, Darmstadt, Germany) staining was performed as previously described [34]. To image BODIPY™ 493/503 fluorescence signals in whole animals, a Zeiss Axio Observer D1 inverted microscope and the filter 38HE (excitation, BP 470/40; beam splitter FT 495; emission BP 525/50) was used. Images were taken by an AxioCam MRm camera (Zeiss, Germany) at fixed exposure times. Objects were magnified using a 20×/0.50 M27 objective. Photographs were taken from 25–50 animals per condition. All experiments were performed four to five times.

### Determination of number and size of lipid droplets

To determine the number and size of lipid droplets, BODIPY™ 493/503 positive structures in pharynx and tail region of L2, L4 and adult staged wild-type animals were imaged using scanning-laser confocal (SLC) microscopy (Leica TCS SP). A HCX PL APO CS 63.0×/1.32 oil immersion objective and Leica LAS AF software was used to collect z-stacks with a step size of 0.5 µm (image format: 158×158 µm^2^, 1024×1024 pixels). Z-stacks consisted of 20 to 110 plane images depending on position and developmental stage of the animal. Images of eight to ten animals per condition and developmental stage were taken using identical settings and exposure times. Z-stacks of pharynx and tail regions were analyzed using ImageJ software (version 1.42q, Object Counter3D plugin). The BODIPY™ 493/503 positive droplets were automatically identified via adaptive thresholding. The volume of each droplet was calculated by summing up the voxels. The total droplet number, total droplet volume and mean droplet volume in pharynx and tail region was calculated using Microsoft Office Exel 2003 software. The volume of BODIPY™ 493/503 positive droplets was classified into different categories: 0–10 µm^3^ (0–2.66 µm in diameter), 10–25 µm^3^ (2.67–3.63 µm in diameter), 25–50 µm^3^ (3.64–4.57 µm in diameter) and >50 µm^3^ (>4.57 µm in diameter).

### Whole genome gene expression analysis

Synchronized wild-type worms cultivated under AL or dDR conditions (dDR0.7, dDR1.5) were harvested at L4 and adult stage. After washing in M9 buffer, worms were resuspended in 350 µl RTL buffer, disrupted in the Precellys 24 homogenizer (Peqlab, Erlangen, Germany) and subjected to an additional homogenization step using QIAshredder spin columns (QIAGEN, Hilden, Germany). Total RNA was isolated using the RNeasy Mini Kit (QIAGEN, Hilden, Germany). On-column DNAase digestion was performed to eliminate genomic DNA (RNase-Free DNase Set, QIAGEN, Hilden, Germany). The quality and yield of the preparation was assessed using a 2100 Bioanalyzer (Agilent Technologies, Waldbronn, Germany).

Labeled cRNA was generated, hybridized and processed by imaGenes expression profiling service (Berlin, Germany) using a customized 8×60 K *C. elegans* Agilent microarrays (imaGenes/SourceBioscience, Steffen Hennig). Normalization was done by ‘quantile normalization’ using the R-package [73]. After normalization, each data set included 26,843 gene expression values of four biological replicates for dDR and AL treated control group. Fold-changes of intensities were calculated from the arithmetic mean of gene expression values between dDR and AL group (L4 stage: dDR1.5vsAL, dDR0.7vsAL; adult stage: dDR1.5vsAL, dDR0.7vsAL). The significance was calculated using an unpaired t-test with unequal variance (Welch-test). P-values>0.05 were regarded as not significant. Genes with a fold-change >2.0 were regarded as differentially regulated.

### Statistical analysis

Statistical analysis was performed with Microsoft Exel (2003) and GraphPad Prism (Version 4.0). Significances were calculated using one-way ANOVA and unpaired t-test (two tailed). Welch-correction was used if variances were different. Logrank t-test was used for lifespan analysis. Differences were considered statistically significant at p<0.05 (*), p<0.01 (**) and p<0.001 (***).

## Supporting Information

Figure S1 Influence of dDR on motility and pumping rate. Body bend frequency (A), whole animal motility (B) and head motility (C) of dDR restricted (dDR1.5 and dDR0.7) and AL fed wild-type worms were analyzed using a worm tracking software (see Experimental procedures). Nematodes were analyzed at first day of adulthood. (A) Results for body bend frequency (Hz) are represented as mean ± SEM of three experiments with 20–30 animals each. (B, C) Bars represent mean motilities (mm s^−1^) ± SEM of three experiments with 20 individuals each (***p<0.001). (D) Mean pharyngeal pumping rate of adult wild-type worms cultivated at either AL or dDR condition (dDR1.5, dDR0.7) was obtained form three experiments with 15–20 individuals each. Error bars represent a SEM. Significant decrease in pharyngeal pumping of dDR1.5 worms is indicated by asterisks (**p<0.01).
(TIF)

Figure S2 Influence of dDR (0.7) in adult N2 worms on the triglyceride (TAG) to phospholipid (PL) ratio determined by thin-layer chromatography (TLC). TAG and PL contents were measured by TLC. Data are shown as mean ± SEM (n = 3). Significant differences to the AL (*ad-libitum*) group were detected using an unpaired two-tailed t-test (*** p<0,001).
(TIF)

Figure S3 Images of fluorescence microscopy (B, D, F) and corresponding bright-field microscopy (A, C, E) of fixative BODIPY 493/503 stained N2 worms at second day of adulthood under AL (A, B), dDR1.5 (C, D) and dDR0.7 (E, F). Representative images of one experiment were shown. Magnification of all photographs 200×.
(TIF)

Figure S4 Images of fluorescence microscopy (B, D, F) and corresponding bright-field microscopy (A, C, E) of fixative BODIPY 493/503 stained N2 worms at eighth day of adulthood under AL (A, B), dDR1.5 (C, D) and dDR0.7 (E, F). Representative images of one experiment were shown. Magnification of all photographs 200×.
(TIF)

Figure S5 Images of fluorescence microscopy of vital BODIPY 493/503 stained N2 worms at first day of adulthood under AL (A) and dDR1.5 (B). green: signals of BODIPY 493/503; blue: signals of autofluorescence lysosome-related organells (LROs). Representative images of three independent experiments were shown. Magnification of all photographs 200×.
(TIF)

Figure S6 Images of fluorescence microscopy of vital BODIPY 493/503 stained N2 wild type (A, B) and *eat-2(ad465)* mutants at L4 larvae stage (A, C) and at adulthood (B, D) under AL condition. Representative images of two independent experiments were shown. Magnification of all photographs 200×.
(TIF)

Figure S7 Images of fluorescence microscopy of vital BODIPY 493/503 stained N2 worms at first day of adulthood under different conditions in order to study the influence of peptone on LD size. A, NGM plates with peptone and a thick bacterial lawn (AL); B, NGM plates without peptone and a thick bacterial lawn (AL condition without peptone); C, dDR6.0 condition. Magnification of all photographs 200×.
(TIF)

Table S1 Body proportion of *C. elegans* cultivated at different dDR conditions.
(DOC)

Table S2 Volume of the maximum-sized lipid droplets under *ad libitum* (AL) and dDR condition.
(DOC)

Table S3 Surface to volume ratio of the mean lipid droplet size under *ad libitum* (AL) and dDR condition.
(DOC)

Table S4 dDR induced alteration of expression of genes implicated in lipid metabolism and storage in *C. elegans*.
(DOC)
