# Supplementary figures and images for: Dietary Restriction during Development Enlarges Intestinal and Hypodermal Lipid Droplets in Caenorhabditis elegans
Source: PLoS One. 2012 Nov 20;7(11):e46198. doi: 10.1371/journal.pone.0046198 (PMC3502458; doi:10.1371/journal.pone.0046198)

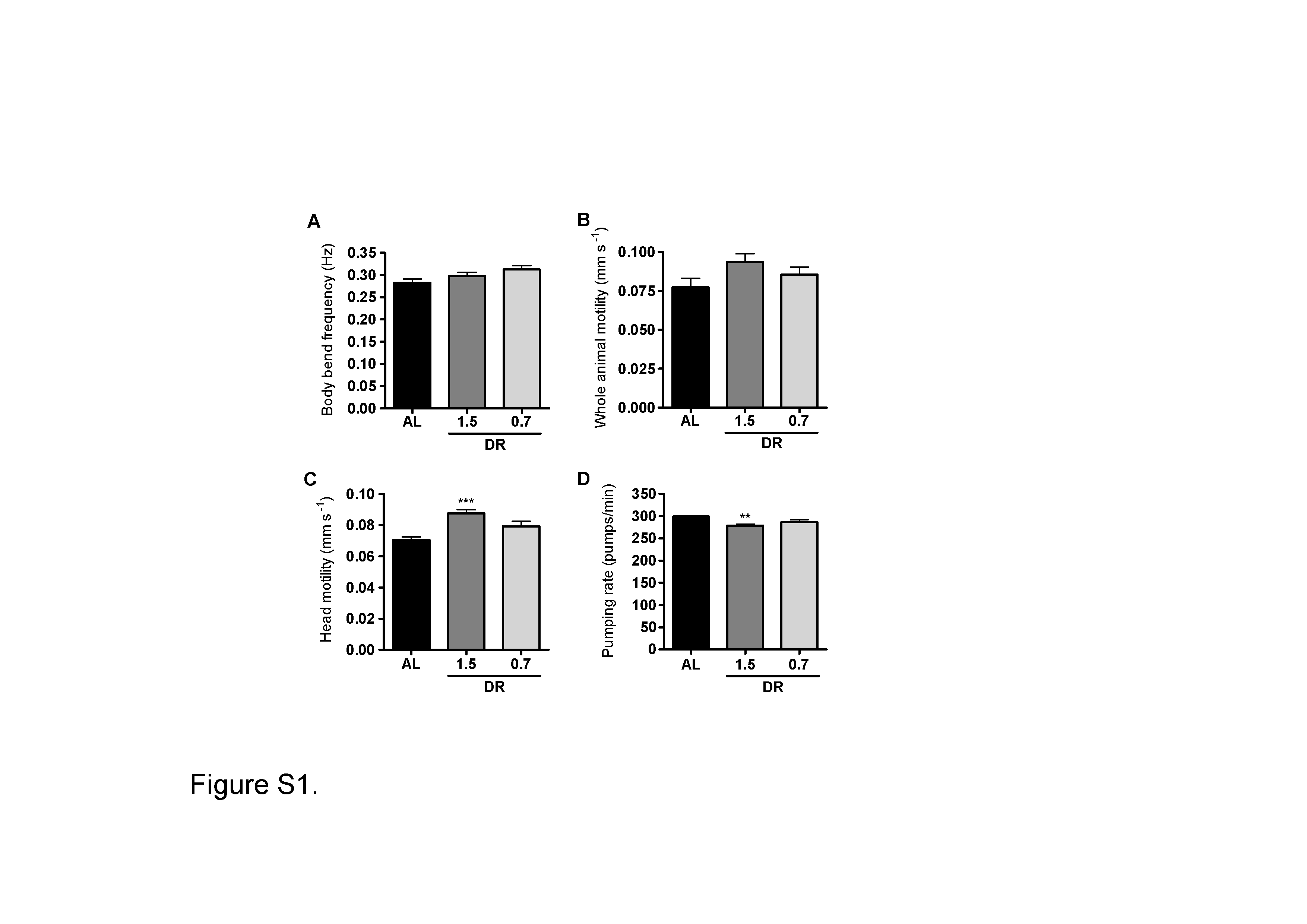

Supplement: Figure S1 — Influence of dDR on motility and pumping rate. Body bend frequency (A), whole animal motility (B) and head motility (C) of dDR restricted (dDR1.5 and dDR0.7) and AL fed wild-type worms were analyzed using a worm tracking software (see Experimental procedures). Nematodes were analyzed at first day of adulthood. (A) Results for body bend frequency (Hz) are represented as mean ± SEM of three experiments with 20–30 animals each. (B, C) Bars represent mean motilities (mm s−1) ± SEM of three experiments with 20 individuals each (***p<0.001). (D) Mean pharyngeal pumping rate of adult wild-type worms cultivated at either AL or dDR condition (dDR1.5, dDR0.7) was obtained form three experiments with 15–20 individuals each. Error bars represent a SEM. Significant decrease in pharyngeal pumping of dDR1.5 worms is indicated by asterisks (**p<0.01). (TIF) [file pone.0046198.s001.tif]

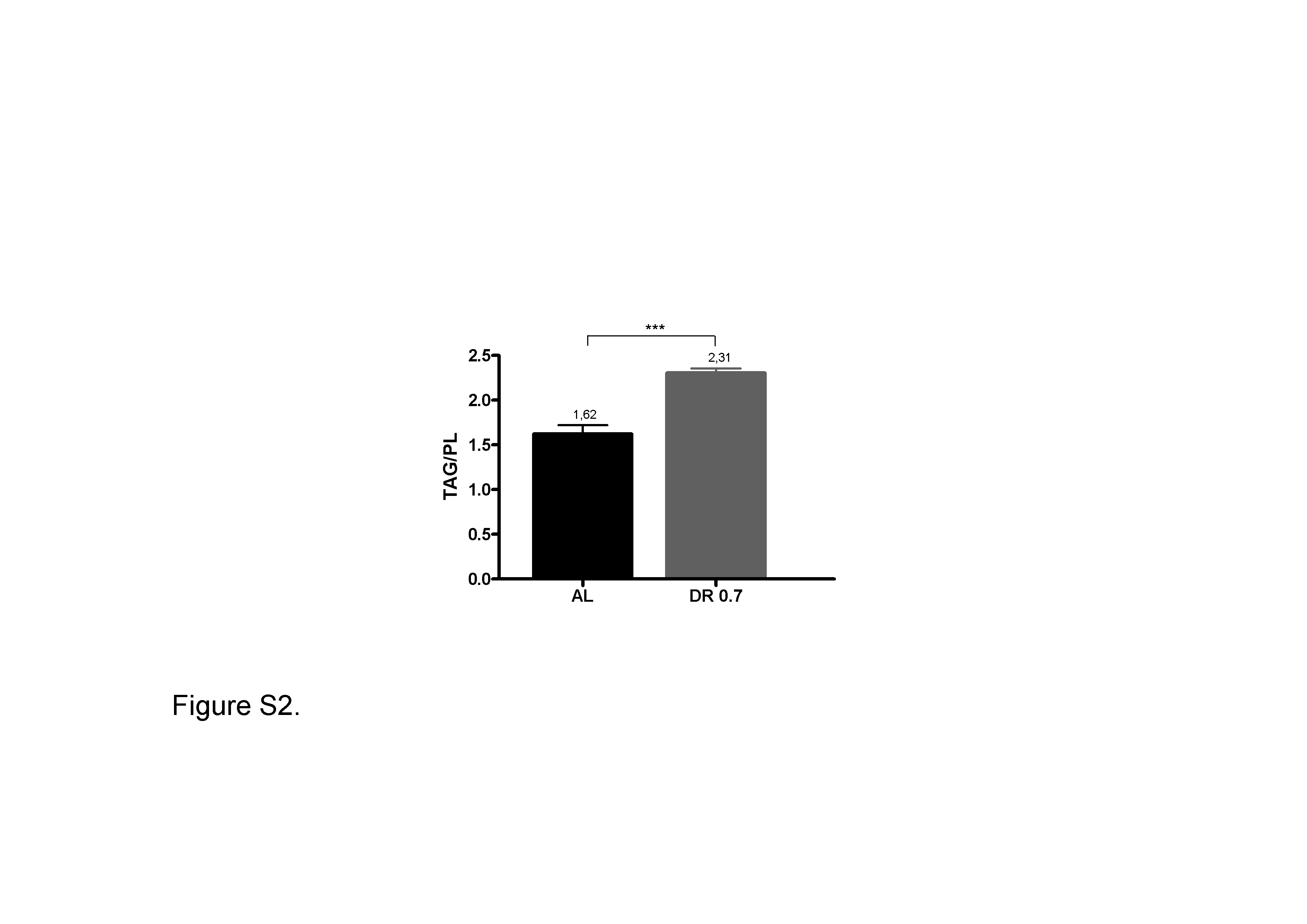

Supplement: Figure S2 — Influence of dDR (0.7) in adult N2 worms on the triglyceride (TAG) to phospholipid (PL) ratio determined by thin-layer chromatography (TLC). TAG and PL contents were measured by TLC. Data are shown as mean ± SEM (n = 3). Significant differences to the AL (ad-libitum) group were detected using an unpaired two-tailed t-test (*** p<0,001). (TIF) [file pone.0046198.s002.tif]

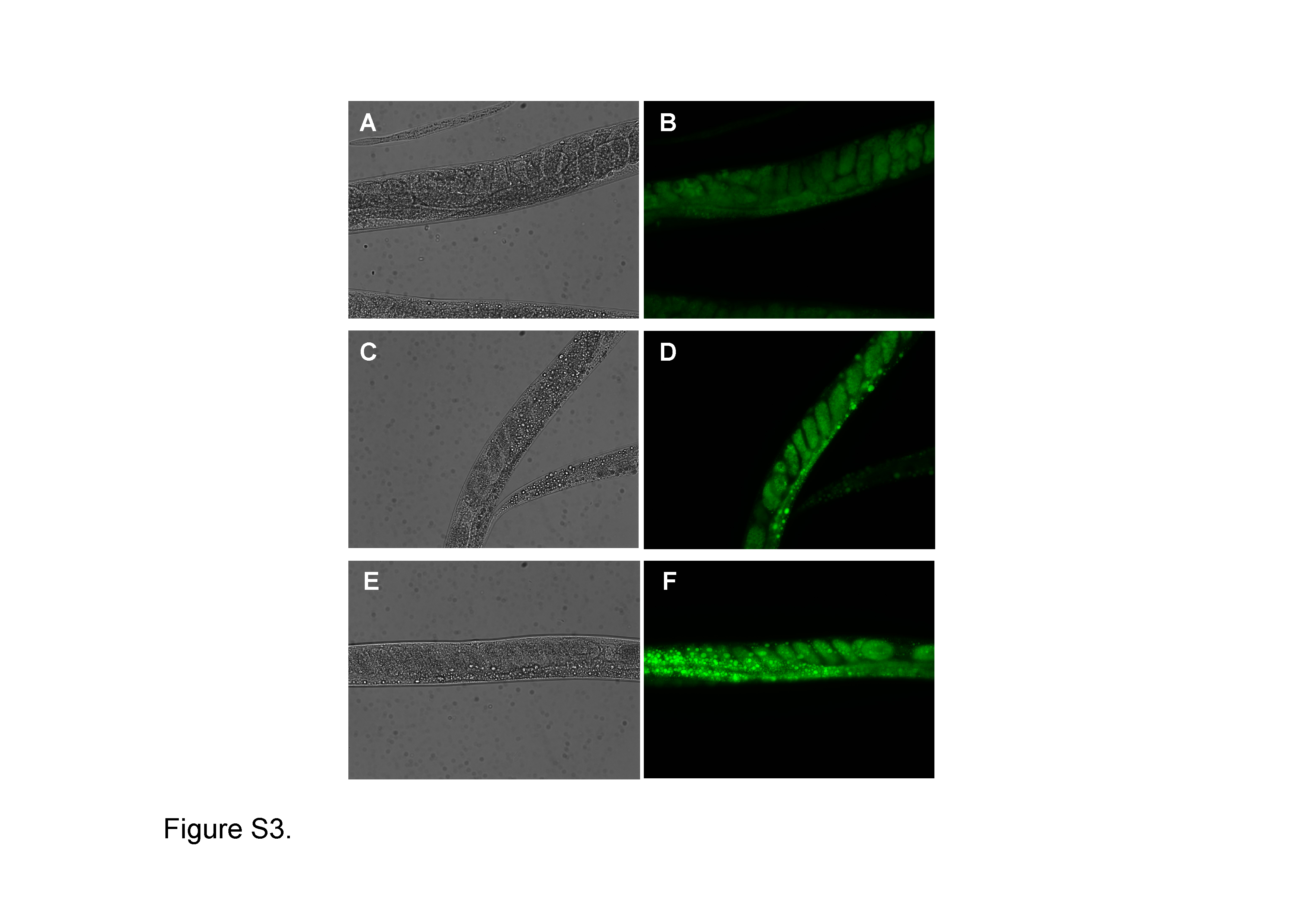

Supplement: Figure S3 — Images of fluorescence microscopy (B, D, F) and corresponding bright-field microscopy (A, C, E) of fixative BODIPY 493/503 stained N2 worms at second day of adulthood under AL (A, B), dDR1.5 (C, D) and dDR0.7 (E, F). Representative images of one experiment were shown. Magnification of all photographs 200×. (TIF) [file pone.0046198.s003.tif]

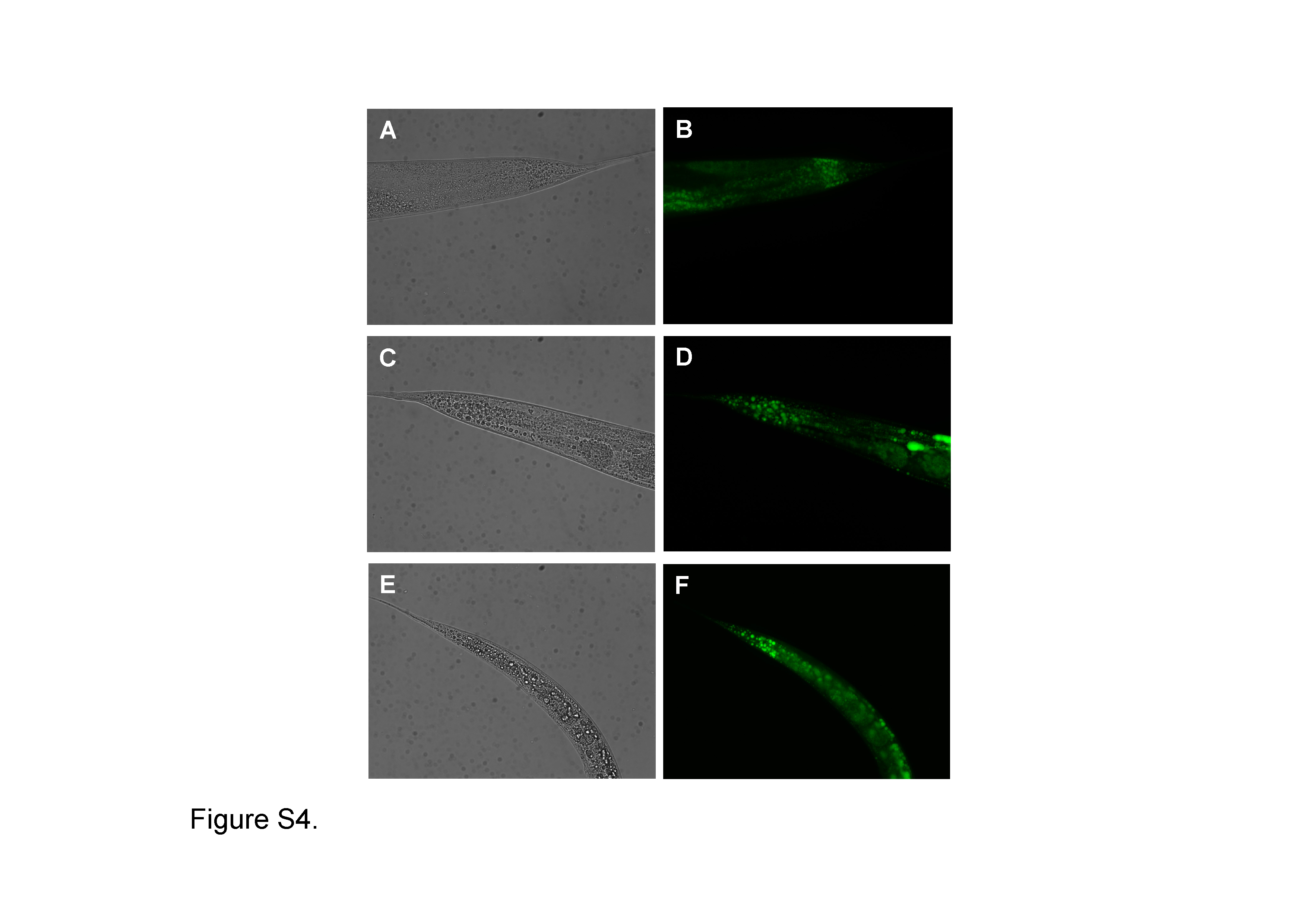

Supplement: Figure S4 — Images of fluorescence microscopy (B, D, F) and corresponding bright-field microscopy (A, C, E) of fixative BODIPY 493/503 stained N2 worms at eighth day of adulthood under AL (A, B), dDR1.5 (C, D) and dDR0.7 (E, F). Representative images of one experiment were shown. Magnification of all photographs 200×. (TIF) [file pone.0046198.s004.tif]

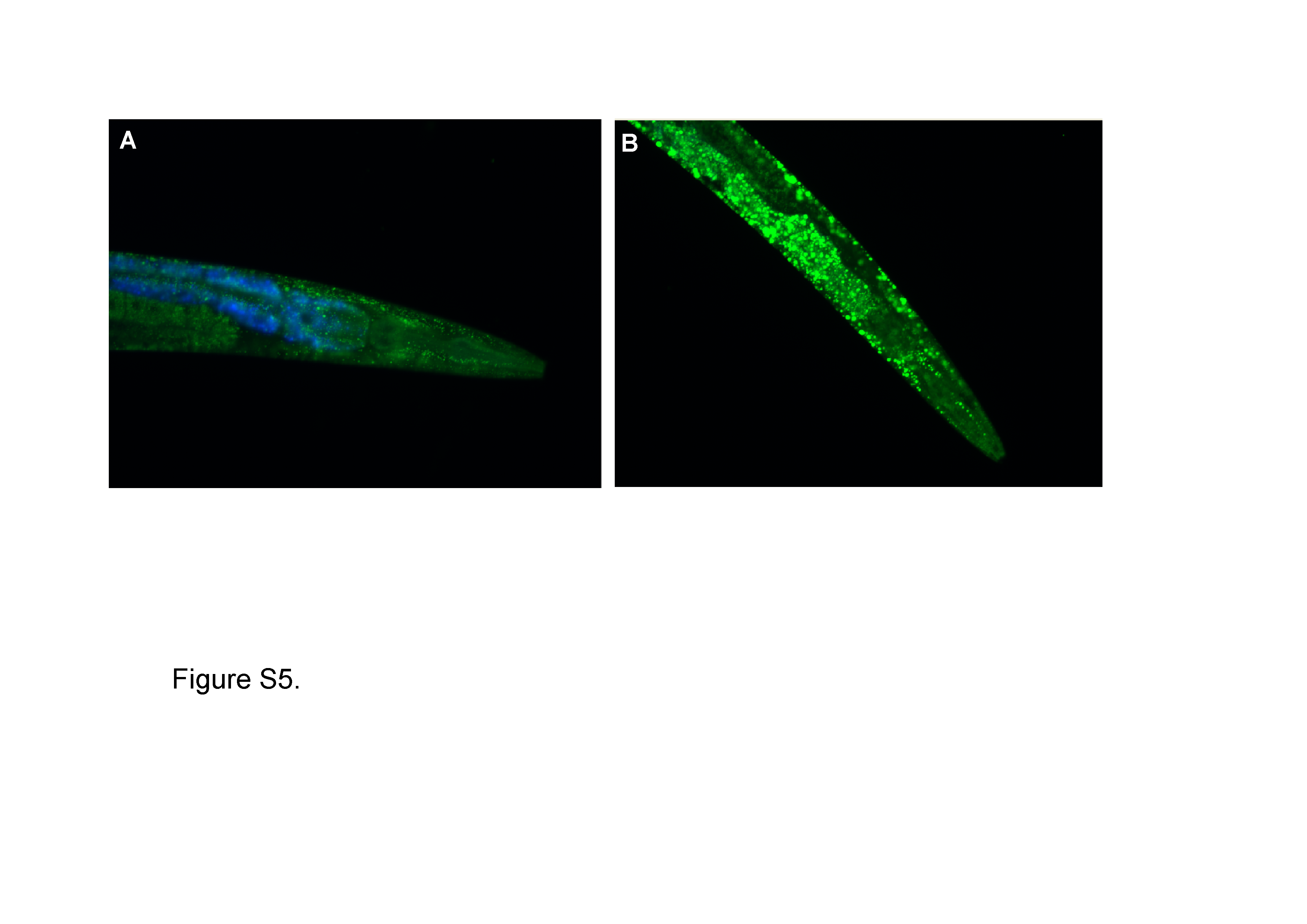

Supplement: Figure S5 — Images of fluorescence microscopy of vital BODIPY 493/503 stained N2 worms at first day of adulthood under AL (A) and dDR1.5 (B). green: signals of BODIPY 493/503; blue: signals of autofluorescence lysosome-related organells (LROs). Representative images of three independent experiments were shown. Magnification of all photographs 200×. (TIF) [file pone.0046198.s005.tif]

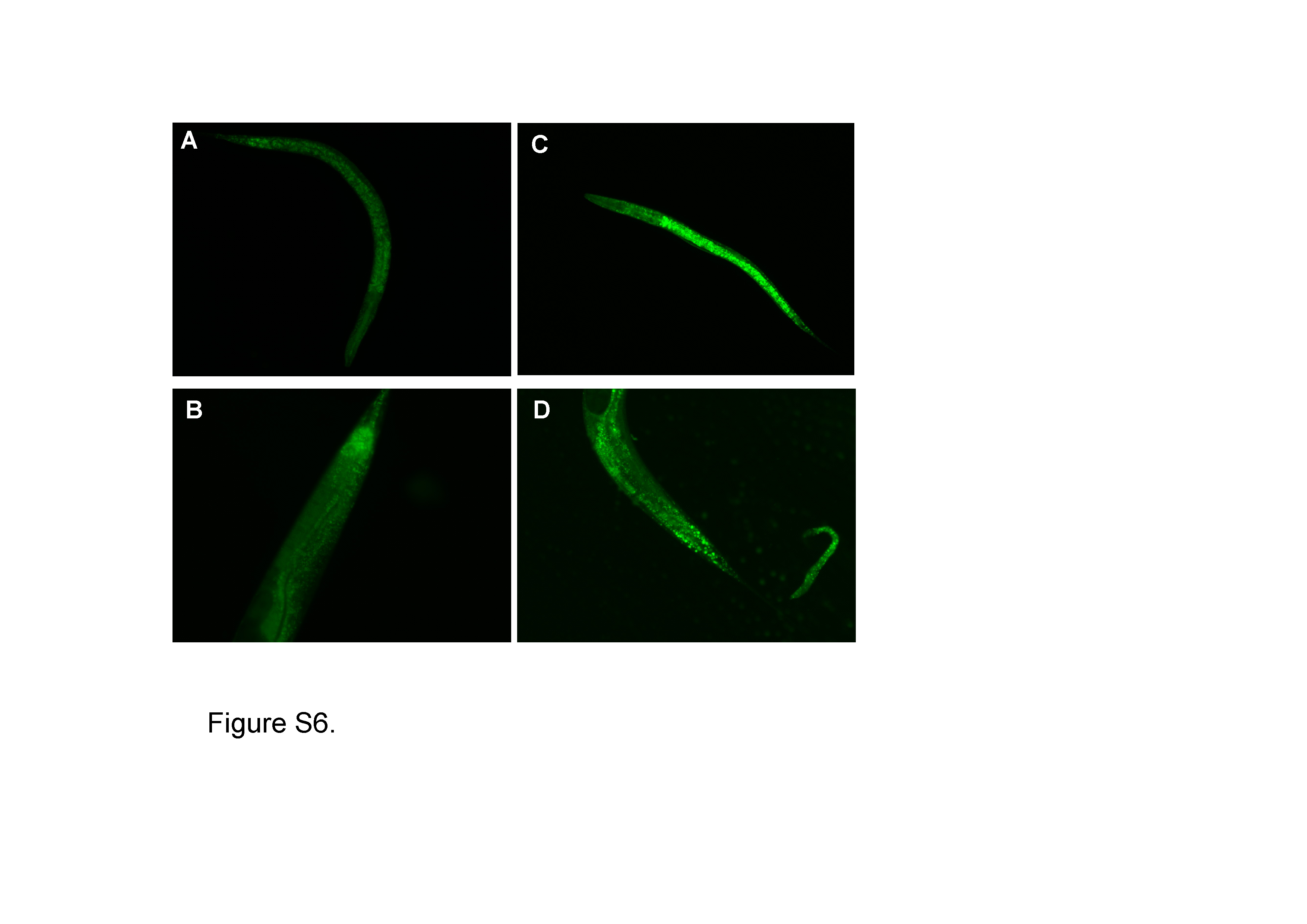

Supplement: Figure S6 — Images of fluorescence microscopy of vital BODIPY 493/503 stained N2 wild type (A, B) and eat-2(ad465) mutants at L4 larvae stage (A, C) and at adulthood (B, D) under AL condition. Representative images of two independent experiments were shown. Magnification of all photographs 200×. (TIF) [file pone.0046198.s006.tif]

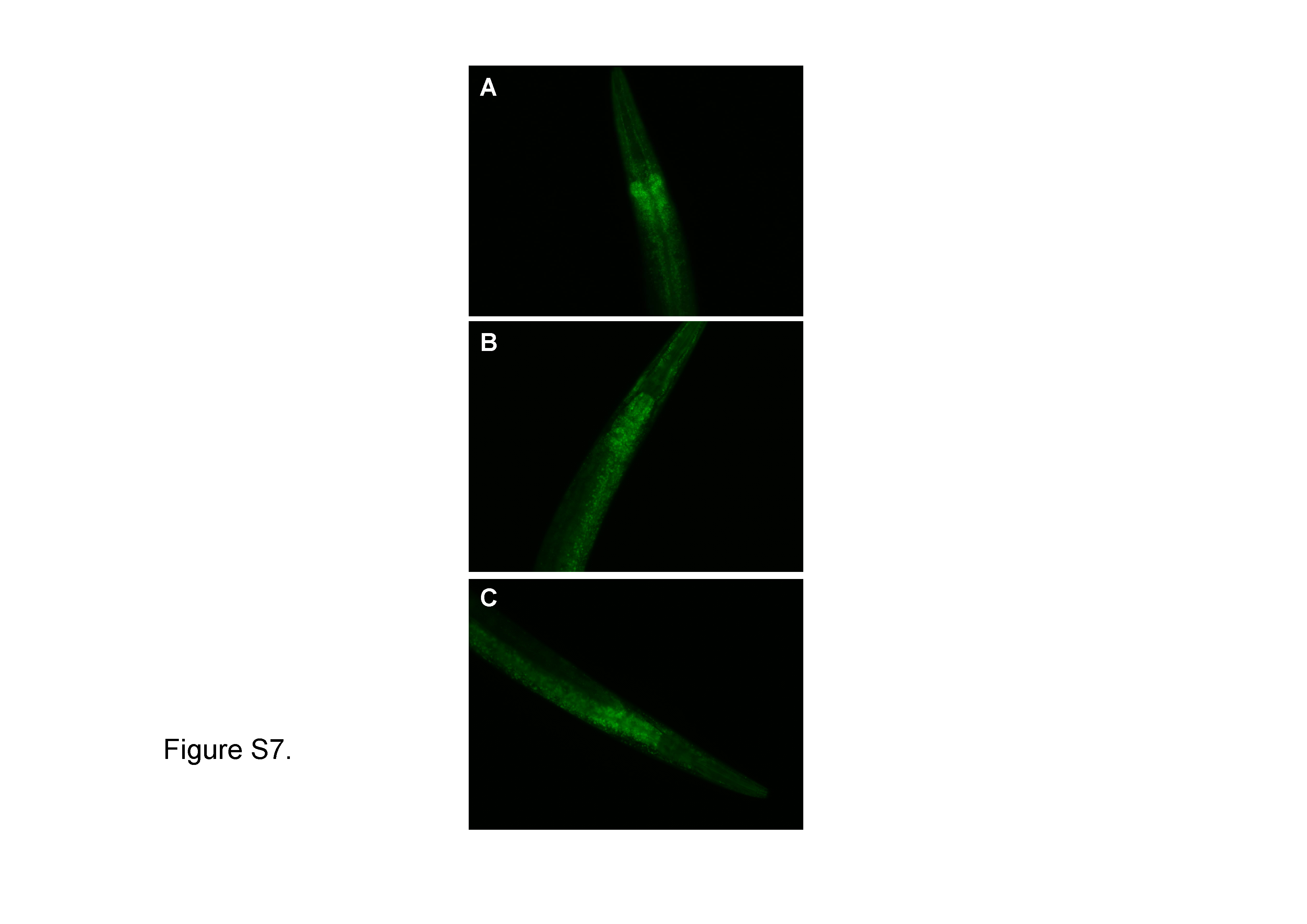

Supplement: Figure S7 — Images of fluorescence microscopy of vital BODIPY 493/503 stained N2 worms at first day of adulthood under different conditions in order to study the influence of peptone on LD size. A, NGM plates with peptone and a thick bacterial lawn (AL); B, NGM plates without peptone and a thick bacterial lawn (AL condition without peptone); C, dDR6.0 condition. Magnification of all photographs 200×. (TIF) [file pone.0046198.s007.tif]
