# Supplementary material for: Dietary Restriction during Development Enlarges Intestinal and Hypodermal Lipid Droplets in Caenorhabditis elegans
Source: PLoS One. 2012 Nov 20;7(11):e46198. doi: 10.1371/journal.pone.0046198 (PMC3502458; doi:10.1371/journal.pone.0046198)
Supplement: Table S1 — Body proportion of C. elegans cultivated at different dDR conditions. (DOC) [file pone.0046198.s008.doc]

**Supporting information – Table S1**

**Table S1. Body proportion of *C. elegans* cultivated at different dietary restriction (dDR) conditions**

| Extent of dDR | Width  (% of AL) | Length  (% of AL) | Volume  (% of AL) |
| --- | --- | --- | --- |
| dDR 6.0 | 100.05 ± 1.78 | 102.94 ±1 .12 | 101.27 ± 3.94 |
| dDR 3.0 | 91.06 ± 1.34 | 97.39 ± 1.11 | 81.88 ± 3.11 |
| dDR 1.5 | 81.22 ± 1.52 | 89.06 ± 1.19 | 60.67 ± 2.95 |
| dDR 1.0 | 79.01 ± 1.38 | 86.72 ± 1.26 | 55.62 ± 2.68 |
| dDR 0.7 | 74.19 ± 1.55 | 80.97 ± 1.40 | 45.86 ± 2.74 |
| dDR 0.3 | 67.82 ± 1.54 | 74.28 ± 1.40 | 34.83 ± 2.08 |

Results for width, length and volume of adult wild-type worms grown at denoted DR conditions are shown as percent of AL treated control group (mean ± SD for AL: width = 77.54 ± 0.94 µm, length = 1274 ± 12.08 µm, volume = 3.76 ± 0.13 nl). Data derived from bright-field microscopy images (see Figure 2). For calculations, the total number of worms per condition was n>50. Results are represented as mean ± SEM of two to three independent experiments.
