# Supplementary material for: Dietary Restriction during Development Enlarges Intestinal and Hypodermal Lipid Droplets in Caenorhabditis elegans
Source: PLoS One. 2012 Nov 20;7(11):e46198. doi: 10.1371/journal.pone.0046198 (PMC3502458; doi:10.1371/journal.pone.0046198)
Supplement: Table S2 — Volume of the maximum-sized lipid droplets under ad libitum (AL) and dDR condition. (DOC) [file pone.0046198.s009.doc]

**Supporting information – Table S2**

**Table S2. Surface to volume ratio of lipid droplets under *ad libitum* (AL) and dDR condition**

|  | **AL** | | **dDR 1.5** | | **dDR 0.7** | |
| --- | --- | --- | --- | --- | --- | --- |
| **stage** | pharynx | tail | pharynx | tail | pharynx | tail |
| **L2** | 4.0 | 4.0 | 3.0 | 2.7 | 3.2 | 2.9 |
| **L4** | 4.0 | 4.1 | 3.2 | 2.8 | 3.0 | 3.1 |
| **adult** | 3.6 | 2.8 | 2.9 | 2.4 | 2.6 | 2.4 |

Dietary restriction (DR1.5, DR0.7) reduces the surface to volume ratio of LDs in comparison to AL condition. The LD surface (µm^2^) was calculated from the mean LD volume (µm3) of all BODIPY 493/503-labeled droplets in pharynx and tail region of L2 larvae, L4 larvae and adult wild-type animals. The mean LD volumes (± SEM) are illustrated in Figure 12.
