# Supplementary material for: Dietary Restriction during Development Enlarges Intestinal and Hypodermal Lipid Droplets in Caenorhabditis elegans
Source: PLoS One. 2012 Nov 20;7(11):e46198. doi: 10.1371/journal.pone.0046198 (PMC3502458; doi:10.1371/journal.pone.0046198)
Supplement: Table S3 — Surface to volume ratio of the mean lipid droplet size under ad libitum (AL) and dDR condition. (DOC) [file pone.0046198.s010.doc]

**Supporting information – Table S3**

**Table S3. Volume of the maximum-sized lipid droplets under *ad libitum* (AL) and dDR condition**

|  | **AL** | | **dDR 1.5** | | **dDR 0.7** | |
| --- | --- | --- | --- | --- | --- | --- |
| **stage** | pharynx | tail | pharynx | tail | pharynx | tail |
| **L2** | 69 ± 12 | 56 ± 6 | 168 ± 25 | 141± 17 | 123 ± 15 | 115 ± 12 |
| **L4** | 66 ± 8 | 54 ± 7 | 128 ± 15 | 113.± 13 | 214 ± 23 | 149 ± 28 |
| **adult** | 58 ± 9 | 213 ± 21 | 235 ± 29 | 609 ± 93 | 285 ± 48 | 406 ± 68 |

Data derive from CLS microscopy images of BODIPY staining wild-type nematodes. The maximum LD volume (µm³) indicates the average size of the five largest LDs detected in respective pharynx and tail region of single animals cultivated at AL and two different DR (DR1.5, DR0.7) conditions. Data are shown as mean ± SEM of eight to ten animals.
