# Supplementary material for: Dietary Restriction during Development Enlarges Intestinal and Hypodermal Lipid Droplets in Caenorhabditis elegans
Source: PLoS One. 2012 Nov 20;7(11):e46198. doi: 10.1371/journal.pone.0046198 (PMC3502458; doi:10.1371/journal.pone.0046198)
Supplement: Table S4 — dDR induced alteration of expression of genes implicated in lipid metabolism and storage in C. elegans. (DOC) [file pone.0046198.s011.doc]

**Supporting information – Table S4**

**Table S4. dDR induced alteration of expression of genes implicated in lipid metabolism and storage in *C. elegans***

|  | **Fold change of regulation^b^** | | | |
| --- | --- | --- | --- | --- |
| **Description^a^** | **L4** | | **adult** | |
|  | **dDR1.5** | **dDR0.7** | **dDR1.5** | **dDR0.7** |
| **Lipases** |  |  |  |  |
| *hosl-1* | 1.2 | ns | 1.0/ -1.1 | -1.2/ -1.3 |
| *atgl-1* | 1.7/ 1.4 | 1.4/ 1.6 | ns | -1.3 |
| *fil-1* | ns | ns | 8.4 | 5.7 |
| *fil-2* | ns | ns | ns | ns |
|  |  |  |  |  |
| **Mitochondrial ß-oxidation** |  |  |  |  |
| Mitochondrial acyl-CoA synthetase |  |  |  |  |
| *acs-3* | 1.1 | 1.1 | 1.8 | 2.0 |
| *acs-11* | ns | 1.6 | 2.5 | 2.8 |
| *acs-13* | ns | ns | -1.3 | -1.1 |
| *acs-15* | -1.4 | -1.7 | 1.7 | 3.5 |
| *acs-18* | -1.4 | - 1.7 | 3.1 | 6.3 |
| R09E10.4 | ns | -2.0 | 1.2 | 2.6 |
|  |  |  |  |  |
| Carnitine palmitoyl transferase I |  |  |  |  |
| *cpt-1* | 1.5 | ns | 1.6 | 1.4 |
| *cpt-3* | 7.3 | ns | 25.7 | 13.6 |
| *cpt-4* | ns | -1.7 | -1.7 | ns |
| *cpt-5* | 2.9 | 2.3 | ns | ns |
| *cpt-6* | 1.5 | ns | ns | -1.4 |
| F41E7.6 | -1.7 | ns | -5.0 | -2.5 |
| F09F3.9 | 2.9 | 2.4 | ns | ns |
|  |  |  |  |  |
| Carnitine palmitoyl transferase II |  |  |  |  |
| *cpt-2* | 1.3 | 1.4 | 1.1 | 1.1 |
|  |  |  |  |  |
| Acyl-CoA dehydrogenase |  |  |  |  |
| *acdh-1* | ns | ns | -1.7 | -1.7 |
| *acdh-2* | ns | -1.7 | -11.0 | -17.5 |
| E04F6.5 | 1.2 | ns | ns | ns |
| F28A10.6 | -1.4 | -1.4 | -2.5 | -3.3 |
| K05F1.3 | -1.4 | -1.4 | 3.0 | 5.7 |
| K06A5.6 | ns | ns | -1.3 | -1.3 |
| T25G12.5 | 1.4 | 1.4 | ns | ns |
|  |  |  |  |  |
| Enoyl-CoA hydratase |  |  |  |  |
| *ech-1* | ns | ns | -1.7 | -2.0 |
| *ech-2* | 1.3 | 1.4 | 1.1 | 1.1 |
| *ech-4* | ns | ns | -2.0 | -2.0 |
| *ech-5* | 0.8 | ns | -1.7 | -2.0 |
| *ech-6* | ns | ns | -2.5 | -2.5 |
| *ech-7* | ns | ns | -2.0 | -2.0 |
|  |  |  |  |  |
| Hydroxy-Acyl-CoA dehydrogenase |  |  |  |  |
| *hacd-1* | ns | ns | 2.6 | 3.8 |
| B0272.3 | 1.4 | 1.3 | 1.3 | 1.3 |
| T08B2.7 | ns | 1.1 | 1.1 | 1.0 |
|  |  |  |  |  |
| Thiolase |  |  |  |  |
| F53A2.7 | ns | ns | -1.4 | -1.4 |
| B0303.3 | 1.1 | ns | ns | ns |
|  |  |  |  |  |
| **Peroxisomal ß-oxidation** |  |  |  |  |
|  |  |  |  |  |
| Acyl-CoA   | 267,788 | | --- | | 236,537 | | 172,533 | | 167,017 | | 151,092 | | 528,699 | | 354,993 | | 294,04 | | 214,744 | | 176,464 | | 1669,348 | | 1280,604 | | 472,029 | | 247,064 | | 197,634 | | 503,96 | | 391,865 | | 235,551 | | 224,097 | | 168,566 | | 272,54 | | 244,798 | | 229,918 | | 173,964 | | 148,85 | | 1573,608 | | 769,131 | | 218,089 | | 214,427 | | 133,688 | | 1986,941 | | 329,938 | | 309,883 | | 227,724 | | 85,844 | | 1992,973 | | 668,608 | | 590,533 | | 471,466 | | 368,946 |   synthetase  *acs-1* | -1.25 | -2.0 | -2.5 | 0.3 |
| *acs-1* | -1.3 | -2.0 | -2.5 | -3.3 |
|  |  |  |  |  |
| Acyl-CoA oxidase |  |  |  |  |
| F08A8.1 | 1.6 | 1.6 | 2.1 | 2.2 |
| F08A8.2 | ns | -1.4 | -2.5 | -2.5 |
| F08A8.3 | 0.8 | -1.3 | -5.0 | -10.0 |
| F08A8.4 | ns | -1.4 | -5.0 | 0.3 |
| F59F4.1 | 1.3 | 1.4 | 1.2 | 1.3 |
| C48B4.1 | ns | ns | -3.3 | -5.0 |
| F25C8.1 | ns | ns | ns | ns |
|  |  |  |  |  |
| Enoyl-CoA hydratase |  |  |  |  |
| *ech-3* | 1.2 | 1.4 | 1.3 | 1.2 |
| *ech-8* | 2.2 | 3.0 | 2.2 | 2.0 |
| *ech-9* | ns | -2.0 | ns | -10.0 |
|  |  |  |  |  |
| Hydratase  *maoc-1* | 2.0 | 2.3 | 1.5 | ns |
|  |  |  |  |  |
| Dehydrogenase  *dhs-28* | 1.5 | 1.7 | 1.5 | 1.5 |
|  |  |  |  |  |
| Thiolase |  |  |  |  |
| *daf-22* | 1.8 | 2.1 | 1.4 | 1.4 |
| T02G5.4 | -3.3 | ns | ns | -1.1 |
| T02G5.7 | -1.3 | -1.4 | ns | -1.1 |
| *kat-1* | -1.1 | ns | ns | -2.0 |
|  |  |  |  |  |
| **Lipid synthesis** |  |  |  |  |
| Fatty acid synthase |  |  |  |  |
| W09B6.1 | ns | ns | 1.6 | 1.7 |
|  |  |  |  |  |
| Acetyl-CoA carboxylase |  |  |  |  |
| F32H2.5 | ns | n.s | n.s | 1.2 |
|  |  |  |  |  |
| Fatty acid desaturase |  |  |  |  |
| *fat-1* | 1.3 | ns | 1.4 | 1.6 |
| *fat-2* | 1.3 | ns | 2.9 | 3.5 |
| *fat-3* | 1.9 | 2.5 | 6.8 | 8.2 |
| *fat-4* | 1.1 | ns | 2.2 | 2.4 |
| *fat-6* | 1.2 | 1.2 | 1.6 | 1.8 |
| *fat-7* | 2.8 | 1.9 | ns | -1.9 |
|  |  |  |  |  |
| Fatty acid elongase |  |  |  |  |
| *elo-1* | 1.2 | ns | 1.3 | 1.2 |
| *elo-2* | ns | ns | ns | ns |
| *elo-3* | -1.7 | -1.7 | -1.7 | -2.0 |
| *elo-4* | -1.3 | -1.3 | ns | ns |
| *elo-5* | ns | -1.7 | -3.3 | -2.5 |
| *elo-6* | ns | ns | -2.0 | -1.7 |
| *elo-7* | -1.3 | -1.7 | 3.3 | 7.7 |
| *elo-8* | -1.5 | -1.6 | 3.2 | 6.2 |
| *elo-9* | ns | ns | ns | ns |
|  |  |  |  |  |
| Diacylglycerol-acyltransferase |  |  |  |  |
| F59A1.10 | ns | ns | -3.3 | -2.5 |
| K07B1.4 | ns | ns | ns | -1.1 |
| *dgtr-1* | ns | -1.1 | -1.4 | -1.7 |
|  |  |  |  |  |
| Phosphatidylcholine synthesis |  |  |  |  |
| *sams-1* | ns | ns | -1.3/ 2.0/ -1.7 | -1.4/ 2.2/ -1.9 |
| *pmt-1* | 1.4/ 1.3 | ns | 4.2/ 4.0 | 4.7/ 4.4 |
| *pmt-2* | 1.5 | ns | -1.9 | -2.1 |
| *pcyt-1* | ns | ns | -2.3 | -2.6 |
|  |  |  |  |  |
| **Lipid transport and storage** |  |  |  |  |
| Fatty acid binding protein |  |  |  |  |
| *lbp-1* | 1.5 | 2.0 | 2.0 | 2.0 |
| *lbp-2* | 2.1 | n.s | n.s | ns |
| *lbp-3* | ns | -1.4 | n.s | ns |
| *lbp-4* | ns | ns | -1.4 | -1.4 |
| *lbp-5* | 1.2 | 1.3 | 1.3 | 1.3 |
| *lbp-6* | 1.2 | ns | -1.7 | -1.7 |
| *lbp-7* | 1.2 | ns | 2.2 | 2.6 |
| *lbp-8* | -1.9 | -2.9 | -33.3 | -40.0 |
|  |  |  |  |  |
| Acyl-CoA binding protein |  |  |  |  |
| *acbp-1* | -1.2 | ns | -1.6 | -1.7 |
| *acbp-3* | ns | ns | -2.0 | -2.0 |
| *acbp-4* | -1.4 | -1.7 | 1.6 | 2.6 |
| *acbp-5* | ns | ns | ns | 1.0 |
| *acbp-6* | -1.7 | -1.9 | -2.6 | -2.9 |
|  |  |  |  |  |
| lipoprotein |  |  |  |  |
| *vit-2* | -1.6 | -2.0 | ns | ns |
| *vit-3* | ns | -3.6 | -1.5 | -2.2 |
| *vit-4* | ns | -4.3 | -1.8 | -3.7 |
| *vit-5* | ns | -2.6 | ns | -1.6 |
| *vit-6* | ns | -1.7/ -2.0 | ns | ns |
|  |  |  |  |  |
| *daf-2* (insulin/IGF receptor ortholog) | ns | 1.6/ 1.3 | 1.2/ -1.9 | 1.2/ -2.3 |
| *daf-7* (transforming growth factor) | 1.3 | 1.5 | ns | ns |
|  |  |  |  |  |
| **Putative lipid droplet associated** |  |  |  |  |
| *cav-2* (caveolin-related) | 1.3 | 1.4 | ns | ns |
| *lpin-1* | 1.2 | 1.3 | 1.2 | 1.1 |
|  |  |  |  |  |
| **Transcription factor** |  |  |  |  |
| *klf-3* | ns | ns | ns | ns |
| *sbp-1* | ns | ns | -1.7 | -1.8 |
| *nhr-49* | 1.7/ 1.5 | 1.6/ 1.6 | 1.3/ 1.2 | 1.2/1.2 |
| *nhr-80* | ns | ns | 1.8/ 1.6 | 1.9/ 1.7 |

This table shows the fold changes of mRNA levels of candidate genes expected being involved in lipid metabolism and lipid storage function in response to DR (DR1.5, DR0.7). ^a^Description of genes is based on the gene ontology (GO) annotation for *C. elegans* (WormBase, www.wormbase.org, release WS 198).

^b^Fold changes are understood between DR and AL group. A positive number indicates a higher gene expression in DR animals. In case of down-regulated genes, the fold change was calculated as 1/ratio, and a minus was added to the quotient. A negative number indicates a lower gene expression under DR. In case of splice variants, fold changes are separated by a slash. Significance threshold was a p-value <0.05. ns, not significant.
